# Effect of Implementing Discharge Readiness Assessment in Adult Medical-Surgical Units on 30-Day Return to Hospital: The READI Randomized Clinical Trial

**DOI:** 10.1001/jamanetworkopen.2018.7387

**Published:** 2019-01-25

**Authors:** Marianne E. Weiss, Olga Yakusheva, Kathleen L. Bobay, Linda Costa, Ronda G. Hughes, Susan Nuccio, Morris Hamilton, Sarah Bahr, Danielle Siclovan, James Bang

**Affiliations:** 1 Marquette University College of Nursing, Milwaukee, Wisconsin; 2 University of Michigan School of Nursing, Ann Arbor; 3 Loyola University Chicago, Marcella Niehoff School of Nursing, Chicago, Illinois; 4 University of Maryland School of Nursing, Baltimore; 5 University of South Carolina College of Nursing, Columbia; 6 Abt Associates, Durham, North Carolina; 7 St Ambrose University Department of Economics, Davenport, Iowa

## Abstract

**Importance:**

The downward trend in readmissions has recently slowed. New enhancements to hospital readmission reduction efforts are needed. Structured assessment of patient readiness for discharge has been recommended as an addition to discharge preparation standards of care to assist with tailoring of risk-mitigating actions.

**Objective:**

To determine the effect of unit-based implementation of readiness evaluation and discharge intervention protocols on readmissions and emergency department or observation visits.

**Design, Setting, and Participants:**

The Readiness Evaluation and Discharge Interventions (READI) cluster randomized clinical trial conducted in medical-surgical units of 33 Magnet hospitals between September 15, 2014, and March 31, 2017, included all adult (aged ≥18 years) patients discharged to home. Baseline and risk-adjusted intent-to-treat analyses used difference-in-differences multilevel logistic regression models with controls for patient characteristics.

**Interventions:**

Of 2 adult medical-surgical nursing units from each hospital, 1 was randomized to the intervention and 1 to usual care conditions. Using the 8-item Readiness for Hospital Discharge Scale, the 33 intervention units implemented a sequence of protocols with increasing numbers of components: READI1, in which nurses assessed patients to inform discharge preparation; READI2, which added patient self-assessment; and READI3, which added an instruction to act on a specified Readiness for Hospital Discharge Scale cutoff score indicative of low readiness.

**Main Outcomes and Measures:**

Thirty-day return to hospital (readmission or emergency department and observation visits). Intervention units above median baseline readmission rate (>11.3%) were categorized as high-readmission units. Among the 33 intervention units, 17 were low-readmission units and 16 were high-readmission units.

**Results:**

The sample included 144 868 patient discharges (mean [SD] age, 59.6 [17.5] years; 51% female; 74 605 in the intervention group and 70 263 in the control group); 17 667 (12.2%) were readmitted and 12 732 (8.8%) had an emergency department visit or observation stay. None of the READI protocols reduced the primary outcome of return to hospital in intent-to-treat analysis of the full sample. In exploratory subgroup analysis, when patient self-assessments were combined with readiness assessment by nurses (READI2), readmissions were reduced by 1.79 percentage points (95% CI, −3.20 to −0.40 percentage points; *P* = .009) on high-readmission units. With nurse assessment alone and on low-readmission units, results were mixed.

**Conclusions and Relevance:**

Implemented in a broad range of hospitals and patients, the READI interventions were not effective in reducing return to hospital. However, adding a structured discharge readiness assessment that incorporates the patient’s own perspective to usual discharge care practices holds promise for mitigating high rates of return to the hospital following discharge.

**Trial Registration:**

ClinicalTrials.gov Identifier: NCT01873118

## Key Points

**Question:**

What is the effect of adding structured nurse assessment of patient readiness for discharge to standard medical-surgical unit discharge practices on 30-day return to hospital?

**Findings:**

In this multisite cluster randomized clinical trial, when patient self-assessments were combined with readiness assessment by nurses, high-readmission units showed a reduction in 30-day hospital returns. Mixed results were observed for nurse assessments only and for low-readmission units.

**Meaning:**

Adding a structured discharge readiness assessment by the discharging nurse that includes patient self-assessment to standard practice for hospital discharge may reduce readmissions and emergency department or observation visits.

## Teaser

This cluster randomized clinical trial investigates the effect of unit-based protocols for ensuring patient readiness for discharge on hospital readmission rates.

## Introduction

With financial penalties from Medicare’s Hospital Readmission Reduction Program affecting three-quarters of hospitals in the United States,^1,2^ hospital systems have prioritized readmission avoidance initiatives.^3,4,5,6^ While discharge transition initiatives generated early successes,^7,8,9,10,11,12^ the downward trend in readmissions has slowed and gaps in readmission rates between high- and low-performing hospitals persist.^13,14^ New enhancements to hospital readmission reduction efforts are needed.

Use of a clinical assessment tool to evaluate patient readiness for discharge has been recommended as an addition to standards of care for discharge preparation.^15,16^ Multiple observational studies indicate that low levels of patient readiness for hospital discharge—as assessed by nurses and patients on the day of discharge—are associated with coping difficulty after discharge and higher likelihood of returning to the hospital for an emergency department (ED) visit or inpatient readmission within 30 days following discharge.^15,16,17,18,19,20,21^ Structured assessment of discharge readiness can assist the health care team in tailoring risk-mitigating actions to patient needs prior to discharge. The aim of the Readiness Evaluation and Discharge Interventions (READI) study was to determine whether implementation of structured discharge readiness assessments during discharge preparation could reduce return to the hospital following discharge.

## Methods

### Study Design

A multisite cluster randomized clinical trial of a unit-level intervention tested, in a 4-phase × 2-condition design, the effect on 30-day return to hospital of adding day-of-discharge readiness assessment protocols, relative to baseline and to usual care control units. The 4 phases included baseline plus 3 READI protocols implemented sequentially with additional components in each phase. The 2 study conditions were intervention (unit incorporated the READI protocols as a supplement to existing discharge practices for all patients going home) and usual care control (unit from the same hospital where no study activities were conducted). Through baseline adjustment, this difference-in-differences design minimized confounding from differences between intervention and control units (such as service lines); by including a usual care control unit, the design also adjusted for any concurrent trends in readmissions unrelated to the intervention (such as system-level readmission reduction efforts).^22,23^

**Figure 1.**
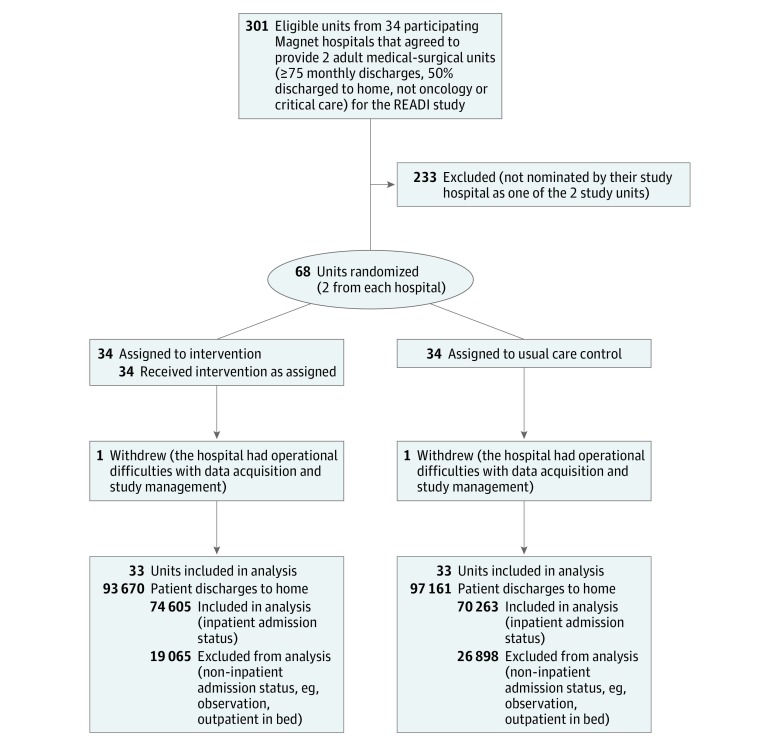
Study Sample Flow Diagram

The study team recruited hospitals through a call for interest to Magnet-designated organizations coordinated by the American Nurse Credentialing Center of the American Nurses Association. Thirty-four hospitals (32 in the United States and 2 in Saudi Arabia) agreed to participate. We assigned 1 unit from each participating hospital as the intervention unit and 1 unit as the control unit using a computer-generated random sequence. One US hospital withdrew because of difficulties with data acquisition and study management; 33 hospitals completed data collection (Figure 1). Approvals were obtained from university institutional review boards of the investigators and participating hospital institutional review boards. For this unit-level implementation, a waiver of patient consent was obtained. We followed the Consolidated Standards of Reporting Trials (CONSORT) guideline for cluster randomized clinical trials. A detailed description of the study protocol, including rationale, design, intervention, study site training, and human subjects and data protections, is included in Supplement 1.

### Intervention: READI Protocols

To uncover optimal protocol components, we incorporated 3 variations of a discharge readiness assessment protocol (labeled READI1, READI2, and READI3) by sequentially adding components into intervention units’ operational procedures for hospital discharge. Each protocol contained a structured assessment of discharge readiness plus an instruction for nurse action. The READI1 protocol required the discharging nurse to complete an assessment of patient readiness on the day of hospital discharge using the Readiness for Hospital Discharge Scale nurse form (RN-RHDS)^16,21^ and instructed the nurses to use their best judgment with the assessment information to guide actions in completing their patients’ preparation for discharge. The READI2 protocol added the patient self-assessment version of the RHDS (PT-RHDS),^16,17^ which was reviewed by the discharging nurse immediately before completing the RN-RHDS, so that the patient’s perspective would inform the nurse’s assessment and action. The READI3 protocol additionally instructed nurses that a score lower than 7 of 10 on the RN-RHDS or PT-RHDS indicates low readiness^16^ and required documentation of an action to improve readiness and reduce readmission risk for any low value.

The RN-RHDS and PT-RHDS are parallel 8-item forms to rate readiness for hospital discharge on the day of discharge on a scale from 0 to 10. Scores below 7 indicate low readiness.^16^ Administered in the 4 hours prior to discharge and following the decision to discharge, the scales measure the degree of readiness for discharge to home self-management close to the time of discharge but with sufficient time to address remaining patient needs before discharge. The forms capture 4 dimensions of readiness (2 items per scale): personal status (physical readiness and energy); knowledge (problems to watch for and restrictions); perceived coping ability (ability to handle demands at home and perform personal care); and expected support (help with personal care and help with medical care).^15^ Scale reliability in prior studies ranged from 0.78 to 0.93.^15,16,17,18,19,20,21^ Nurses can complete the form in under 2 minutes; patients typically require 2 to 5 minutes. The RN-RHDS and PT-RHDS forms are included in the study protocol (eAppendix in Supplement 2).

Intervention unit nurses attended mandatory training in the 2 weeks prior to the start of each protocol that included an overview of prior research on discharge readiness, the association with postdischarge outcomes, and detailed instructions on implementation of the protocols. Training in the study protocol was incorporated into orientation for newly hired nurses, and training modules were available for float nurses who were assigned patients being discharged. Units developed logistical plans to restrict cross-assignment of float nurses between intervention and control units. Information about the study protocol was withheld from control and other hospital units. Control units used their established discharge practices during the entire study.

### Sample Criteria

We defined eligible inpatient units as adult medical, surgical, or medical-surgical units with a minimum of 75 discharges per month of which at least 50% were discharged to home. We excluded critical care and oncology units. Eligible patients were adults (aged ≥18 years) discharged to home following an inpatient admission. Patients not admitted on inpatient status (observation or short stay) were excluded. Eligibility at discharge was derived from discharge disposition codes in the electronic health record: home with self-care, home with home health, home with hospice, or left against medical advice (Figure 1).

### Sample Size Estimation

A multilevel nested patient sample (hospitals, units, patients) was calculated for a baseline-adjusted cluster randomized design. After oversampling to account for clustering at the unit and hospital level, we estimated the minimum sample size required to achieve 80% power and 5% 2-tailed significance for a small effect size on readmissions (0.02 change in pseudo-*R*^2^) in subgroup analyses to be 24 304 patients (90 per unit per phase).^24^ Each protocol phase was 4 months in duration to ensure the accrual of the targeted patient sample for each protocol on small units (approximately 75 eligible monthly discharges), assuming 50% patient eligibility.

### Study Period and Data Collection

Each site had a total data collection period of 17.5 months between September 15, 2014, and March 31, 2017 (baseline and 3 READI protocol phases of 4 months each, and 3 two-week training periods). We extracted patient and encounter characteristics, including outcomes for return to the hospital following discharge, from study sites’ electronic health records at least 120 days after the end of each phase. The RN-RHDS and PT-RHDS forms were either completed on paper or through study sites’ electronic health records. Paper forms were scanned and merged with electronic data files.

### Study Outcomes and Measures

The primary outcome was all-cause, same-hospital return visits within 30 days following discharge. A return visit was defined as a categorical variable, with reference category 0 = no return: no record of readmissions, ED visits, or observation stays (not admitted with inpatient status, coded as short stay <23 hours, outpatient in bed); 1 = ED/Obs: no record of readmission and at least 1 ED visit or observation stay (ED and observation were combined as return to the hospital without inpatient readmission); and 2 = readmission (≥1 inpatient readmission). 

We risk adjusted for patient factors previously found to be associated with return visits.^25^ Socioeconomic factors included age, sex, race/ethnicity, marital status, and payer type. Clinical factors included Major Diagnostic Category (MDC),^26^ admission type (medical vs surgical), prior hospitalization within 30 and 90 days, length of stay, intensive care unit stay, Elixhauser Comorbidity Index,^27^ and discharge disposition. Among the 33 intervention units, 17 were low-readmission units and 16 were high-readmission units.

### Statistical Analysis

We estimated the intent-to-treat (ITT) effect using the full population of eligible discharges during the intervention period. We also report treated-per-protocol (TPP) results that excluded patients on intervention units who did not receive a discharge readiness assessment per 1 of the READI protocols.^28^

An encounter-level conditional likelihood difference-in-differences multivariate logistic regression model that adjusted for multiple comparisons was used to test the effect of the intervention for each protocol. The difference-in-differences approach^22,23^ was modeled as a 4 × 2 set of interaction terms between the study protocol phases (baseline, READI1, READI2, and READI3) and study conditions (intervention and control). This allowed for a simultaneous estimation of (1) changes in readmission and ED/Obs visits during the 3 sequential protocol phases relative to baseline (first differences) and (2) differences in the magnitudes of these changes between the intervention and the control units (second differences, or difference-in-differences). If any of the READI protocols were effective in reducing return to the hospital (beyond any possible reductions from system-level efforts or any other unrelated spurious effects), the difference-in-differences estimates will show a significantly greater magnitude of the reduction on the intervention units relative to the control units. We estimated predictive margins for the difference-in-differences effects for each protocol and tested for significance using a 2-sided Wald test at the standard 5% significance level (*P* < .05). (See eAppendix in Supplement 2 for a detailed description of the difference-in-differences approach used for this study.)

For 2 study sites that did not report observation stays, we imputed missing values for observation stays for each patient using multiple imputation with a logistic model and patient and encounter characteristics. Missing values on patient characteristics were not missing at random and were coded as a separate category for each variable.

We adjusted for patient factors and hospital effects and used robust standard errors to account for data grouping at the hospital and unit levels. We used a finite population correction to adjust for sampling without replacement from a finite population of hospital units.

To explore differential effectiveness for high- and low-readmission units, we stratified high- and low-readmission groups on completion of data collection based on unadjusted baseline 30-day readmission rates for the intervention units. We defined units above the median readmission rate on the intervention units at baseline (11.3%) as high-readmission units; units at or below the median baseline readmission rate were categorized as low-readmission units. Classification of units into high- and low-readmission categories for stratified analyses occurred after the data collection phase of the study was complete, thus minimizing allocation and treatment bias. We used a moderated analysis model that interacted the primary specification with each unit’s high- or low-readmission category. Analyses were performed using Stata version 15.1 statistical software (StataCorp).

## Results

### Sample Characteristics

A total of 144 868 patients (mean [SD] age, 59.6 [17.5] years; 51% female) were eligible for the study (74 605 in the intervention group and 70 263 in the control group) (Figure 1). For the 3 protocols, 70.84% of intervention unit patients (39 176 of 55 301) were treated per protocol (READI1: 73.88% [13 806 of 18 867]; READI2: 71.84% [13 940 of 19 403]; and READI3: 66.41% [11 430 of 17 211]). Table 1 presents sample size by protocol phase and study condition.

**Table 1.** Patient-Level Characteristics

Characteristic	All (N = 144 868)	Control Units (n = 70 263)	Intervention Units	
			ITT Sample^a^ (n = 74 605)	TPP Sample^b^ (n = 39 176)
Baseline, No.	37 323	18 019	19 304	0
READI1, No.	36 736	18 049	18 687	13 806
READI2, No.	37 848	18 445	19 403	13 940
READI3, No.	32 961	15 750	17 211	11 430
Length of stay, mean (SD), d	4.50 (5.42)	4.42 (5.62)	4.58 (5.22)	4.68 (5.48)
Age, mean (SD), y	59.59 (17.54)	60.32 (17.37)	58.91 (17.67)	58.33 (17.50)
Elixhauser Comorbidity Index, mean (SD)	6.60 (8.25)	6.59 (8.27)	6.61 (8.23)	6.55 (8.24)
Sex, No. (%)				
Male	70 679 (48.8)	34 448 (49.0)	36 231 (48.6)	19 122 (48.8)
Female	74 189 (51.2)	35 815 (51.0)	38 374 (51.4)	20 054 (51.2)
Race, No. (%)				
American Indian or Alaska Native	1206 (0.8)	493 (0.7)	713 (1.0)	430 (1.1)
Asian	4737 (3.3)	2526 (3.6)	2211 (3.0)	990 (2.5)
Black or African American	21 009 (14.5)	10 901 (15.5)	10 108 (13.5)	5199 (13.3)
Hawaiian or Pacific Islander	452 (0.3)	257 (0.4)	195 (0.3)	82 (0.2)
White	94 191 (65.0)	45 111 (64.2)	49 080 (65.8)	25 782 (65.8)
Unknown	23 273 (16.1)	10 975 (15.6)	12 298 (16.5)	6693 (17.1)
Ethnicity, No. (%)				
Non-Hispanic and non-Latino	121 001 (83.5)	59 025 (84.0)	61 976 (83.1)	31 229 (79.7)
Hispanic or Latino	21 696 (15.0)	10 153 (14.5)	11 543 (15.5)	7151 (18.3)
Unknown	2171 (1.5)	1085 (1.5)	1086 (1.5)	796 (2.0)
Marital status, No. (%)				
Not married	62 224 (43.0)	29 837 (42.5)	32 387 (43.4)	17 198 (43.9)
Married	67 513 (46.6)	32 034 (45.6)	35 479 (47.6)	18 894 (48.2)
Unknown	15 131 (10.4)	8392 (11.9)	6739 (9.0)	3084 (7.9)
Payer type, No. (%)				
Private	42 511 (29.3)	19 681 (28.0)	22 830 (30.6)	12 140 (31.0)
Medicare	58 307 (40.2)	29 267 (41.7)	29 040 (38.9)	14 685 (37.5)
Medicaid	20 016 (13.8)	9780 (13.9)	10 236 (13.7)	5113 (13.1)
Uninsured	3429 (2.4)	1576 (2.2)	1853 (2.5)	787 (2.0)
Other	20 605 (14.2)	9959 (14.2)	10 646 (14.3)	6451 (16.5)
Intensive care unit stay, No. (%)				
No	119 035 (82.2)	58 262 (82.9)	60 773 (81.5)	31 822 (81.2)
Yes	25 833 (17.8)	12 001 (17.1)	13 832 (18.5)	7354 (18.8)
Major Diagnostic Category, No. (%)				
Nervous system	8540 (5.9)	4495 (6.4)	4045 (5.4)	2035 (5.2)
Eye	246 (0.2)	139 (0.2)	107 (0.1)	59 (0.2)
Ear, nose, and throat	1871 (1.3)	853 (1.2)	1018 (1.4)	546 (1.4)
Respiratory	16 217 (11.2)	7211 (10.3)	9006 (12.1)	4600 (11.7)
Circulatory	28 248 (19.5)	16 478 (23.5)	11 770 (15.8)	6046 (15.4)
Digestive	19 927 (13.8)	8239 (11.7)	11 688 (15.7)	5970 (15.2)
Hepatobiliary and pancreatic	7389 (5.1)	3131 (4.5)	4258 (5.7)	2199 (5.6)
Musculoskeletal	10 579 (7.3)	6111 (8.7)	4468 (6.0)	2278 (5.8)
Skin and subcutaneous	4368 (3.0)	2033 (2.9)	2335 (3.1)	1233 (3.1)
Endocrine and metabolic	6767 (4.7)	2552 (3.6)	4215 (5.6)	2282 (5.8)
Kidney and urinary	9127 (6.3)	4494 (6.4)	4633 (6.2)	2469 (6.3)
Male reproductive	956 (0.7)	547 (0.8)	409 (0.5)	223 (0.6)
Female reproductive	2400 (1.7)	1205 (1.7)	1195 (1.6)	647 (1.7)
Pregnancy	801 (0.6)	417 (0.6)	384 (0.5)	160 (0.4)
Blood and immunological	2838 (2.0)	1120 (1.6)	1718 (2.3)	869 (2.2)
Myeloproliferative	700 (0.5)	407 (0.6)	293 (0.4)	149 (0.4)
Infectious and parasitic	9864 (6.8)	4614 (6.6)	5250 (7.0)	2738 (7.0)
Mental	311 (0.2)	154 (0.2)	157 (0.2)	60 (0.2)
Alcohol and drug	1699 (1.2)	715 (1.0)	984 (1.3)	517 (1.3)
Injury, poison, and toxin	2146 (1.5)	1004 (1.4)	1142 (1.5)	574 (1.5)
Multiple trauma	316 (0.2)	166 (0.2)	150 (0.2)	76 (0.2)
HIV	224 (0.2)	103 (0.1)	121 (0.2)	67 (0.2)
Transplants	261 (0.2)	72 (0.1)	189 (0.3)	106 (0.3)
Unrelated	967 (0.7)	498 (0.7)	469 (0.6)	254 (0.6)
Other	757 (0.5)	375 (0.5)	382 (0.5)	195 (0.5)
Missing	7349 (5.1)	3130 (4.5)	4219 (5.7)	2824 (7.2)
Patient type, No. (%)				
Medical	100 692 (69.5)	49 043 (69.8)	51 649 (69.2)	26 313 (67.2)
Surgical	41 001 (28.3)	20 359 (29.0)	20 642 (27.7)	11 170 (28.5)
Unknown	3175 (2.2)	861 (1.2)	2314 (3.1)	1693 (4.3)
Hospitalization in prior 30 d, No. (%)				
No	108 981 (75.2)	52 282 (74.4)	56 699 (76.0)	30 635 (78.2)
Yes	17 514 (12.1)	7920 (11.3)	9594 (12.9)	5289 (13.5)
Unknown	18 373 (12.7)	10 061 (14.3)	8312 (11.1)	3252 (8.3)
Hospitalization in prior 90 d, No. (%)				
No	98 656 (68.1)	47 472 (67.6)	51 184 (68.6)	27 906 (71.2)
Yes	28 971 (20.0)	13 305 (18.9)	15 666 (21.0)	8488 (21.7)
Unknown	17 241 (11.9)	9486 (13.5)	7755 (10.4)	2782 (7.1)
30-d outcomes: return to hospital, No. (%)				
None	114 469 (79.0)	55 398 (78.8)	59 071 (79.2)	31 225 (79.7)
ED/Obs^c^	12 732 (8.8)	6153 (8.8)	6579 (8.8)	3421 (8.7)
Readmission	17 667 (12.2)	8712 (12.4)	8955 (12.0)	4530 (11.6)

Abbreviations: ED, emergency department; ITT, intent-to-treat; Obs, observation; READI, Readiness Evaluation and Discharge Interventions Protocol; TPP, treated-per-protocol.

^a^ Includes patients in baseline, READI1, READ2, and READI3 protocols.

^b^ Includes patients in READI1, READi2, and READI3 who were treated per protocol. Baseline patients on intervention units were excluded.

^c^ Emergency department visit or observation stay with no record of readmission. Emergency department and observation were combined as return to hospital without inpatient readmission.

The hospitals were geographically diverse and represented a mix of community, urban, and academic medical centers. Intervention and control units were similar in number of licensed beds, baseline readmission rates, and presence of discharge transition programs (Table 2). Patients on intervention and control units had similar sociodemographic and clinical characteristics, with some variation in major diagnostic categories owing to the patient populations of the units selected for the study. There were 17 667 readmissions and 12 732 ED/Obs visits without a readmission within 30 days following discharge, corresponding to a 12.2% unadjusted readmission rate and 8.8% unadjusted ED/Obs visit rate in the study sample (Table 1).

**Table 2.** Hospital and Unit Characteristics

Characteristic	No. (%)		
	Total Units (N = 66 Units)^a^	Low-Readmission Intervention Units (n = 17 of 33 Units)	High-Readmission Intervention Units (n = 16 of 33 Units)
**Hospital-Level Characteristics**			
Licensed beds, mean (SD), No.	546.97 (336.46)	441.76 (212.62)	658.75 (409.16)
Geographic location			
Northeast United States	14 (42.4)	5 (29.4)	9 (56.3)
Midwest United States	8 (24.2)	4 (23.5)	4 (25.0)
North United States	2 (6.1)	2 (11.8)	0
West or Southwest United States	5 (15.2)	2 (11.8)	3 (18.8)
South United States	2 (6.1)	2 (11.8)	0
Saudi Arabia	2 (6.1)	2 (11.8)	0
Teaching status			
Community			
Nonteaching	12 (36.4)	8 (47.1)	4 (25.0)
Teaching	7 (21.2)	2 (11.8)	5 (31.3)
Urban			
Nonteaching	4 (12.1)	1 (5.9)	3 (18.8)
Teaching	4 (12.1)	4 (23.5)	0
Academic medical center	6 (18.2)	2 (11.8)	4 (25.0)
**Unit-Level Characteristics**			
Intervention units			
Licensed beds, mean (SD), No.	36.06 (11.78)	37.18 (9.57)	34.87 (13.99)
30-d rate per 100 discharges at baseline, mean (SD), %			
Readmission	12.08 (3.50)	9.92 (2.94)	14.39 (2.42)
ED/Obs^b^	9.00 (2.70)	8.59 (3.31)	9.42 (1.85)
Type			
Medical	7 (21.2)	1 (5.9)	6 (37.5)
Surgical	3 (9.1)	3 (17.6)	0
Medical-surgical combined	15 (45.5)	8 (47.1)	7 (43.8)
Step-down	7 (21.2)	4 (23.5)	3 (18.8)
Blended acuity	1 (3.0)	1 (5.9)	0
Discharge model^c^			
National	5 (15.2)	2 (12.5)	4 (23.5)
State, local, or other	11 (33.3)	6 (37.5)	5 (29.4)
None	17 (51.5)	8 (50.0)	8 (47.1)
Control units			
Licensed beds, mean (SD), No.	33.00 (9.53)	34.29 (10.61)	31.63 (8.38)
30-d rate per 100 discharges at baseline, mean (SD), %			
Readmission	12.28 (4.34)	10.16 (3.62)	14.53 (2.42)
ED/Obs^b^	8.92 (3.14)	8.63 (3.83)	9.23 (2.26)
Type			
Medical	10 (30.3)	4 (23.5)	6 (37.5)
Surgical	4 (12.1)	3 (17.6)	1 (6.3)
Medical-surgical combined	13 (39.4)	6 (35.3)	7 (43.8)
Step-down	6 (18.2)	4 (23.5)	2 (12.5)
Blended acuity	0	0	1 (5.9)
Discharge model^c^			
National	6 (18.2)	1 (6.3)	4 (23.5)
State, local, or other	13 (39.4)	6 (37.5)	7 (41.2)
None	14 (42.4)	9 (56.3)	6 (35.3)

Abbreviations: ED, emergency department; Obs, observation.

^a^ Includes 33 hospitals each with 1 intervention unit and 1 control unit.

^b^ Emergency department visit or observation stay with no record of readmission. We combined ED and Obs as return to the hospital without inpatient readmission.

^c^ National initiatives include Transitional Care Model, Care Transition Model, RED, BOOST, IHI/State Action on Avoidable Readmissions, IHI/American College of Cardiology Hospital to Home (H2H), and Centers for Medicare & Medicaid Services Care Transition Project: Interventions to Reduce Acute Care Transfers (INTERACT). State, local, or other includes state hospital association and other regional collaboratives and local or hospital-specific initiatives.

Mean scores on RN-RHDS and PT-RHDS increased across the protocol phases: RN-RHDS increased from 8.14 (out of 10) during READI1 to 8.20 with READI2 (difference, 0.06; 95% CI, −0.04 to 0.16; *P* = .23) and to 8.60 (difference, 0.46, 95% CI, 0.29-0.64; *P* < .001) with READI3; PT-RHDS increased from 8.42 during READI2 to 8.64 (difference, 0.23; 95% CI, −0.11 to 0.35; *P* < .01) with READI3. A total of 15.7% of patients scored less than 7 on nurse assessments and 12.5% scored less than 7 on patient assessments.

### Intervention Effect on Outcomes

The ITT analysis for the full sample revealed a small significant increase in readmissions by 1.02 absolute percentage point change (95% CI, 0.10-1.90 percentage points; *P* = .03) and a concurrent reduction in ED/Obs visits by a similar magnitude (−1.15 percentage points; 95% CI, −2.00 to −0.30 percentage points; *P* = .008) with the READI1 protocol. The READI2 and READI3 protocols did not significantly affect readmissions and ED/Obs visits. The TPP analysis revealed no significant changes in readmissions or ED visits for any of the protocols (Table 3).

**Table 3.** Difference-in-Differences Estimates of the Effect of the READI Intervention on 30-Day Return to the Hospital Following Discharge

Return to Hospital	READI1^a^				READI2^b^				READI3^c^			
	Intervention, Adjusted Rate (95% CI)^d^	Control, Adjusted Rate (95% CI)^d^	Difference (95% CI)	*P* Value	Intervention, Adjusted Rate (95% CI)^d^	Control, Adjusted Rate (95% CI)^d^	Difference (95% CI)	*P* Value	Intervention, Adjusted Rate (95% CI)^d^	Control, Adjusted Rate (95% CI)^d^	Difference (95% CI)	*P* Value
**Intent to Treat**												
Readmission	12.94 (12.14 to 14.75)	11.92 (11.46 to 12.39)	1.02 (0.10 to 1.90)	.03	12.23 (11.46 to 12.99)	12.43 (1.96 to 12.90)	−0.2 (−1.10 to 0.70)	.66	12.23 (11.76 to 12.69)	11.64 (11.14 to 12.13)	0.59 (−0.30 to 1.50)	.22
ED/Obs^e^	8.45 (7.76 to 9.15)	9.6 (9.18 to 10.20)	−1.15 (−2.0 to −0.30)	.008	8.39 (7.69 to 9.10)	8.69 (8.29 to 9.09)	−0.29 (−1.10 to 0.50)	.47	8.71 (8.29 to 9.14)	9.35 (8.90 to 9.79)	−0.64 (−1.50 to 0.20)	.14
**Treated per Protocol**												
Readmission	12.27 (11.11 to 13.42)	11.83 (11.20 to 12.46)	0.44 (−0.80 to 1.60)	.46	11.32 (1.54 12.10)	12.45 (11.58 13.31)	−1.13 (−2.40 to 0.20)	.08	12.26 (11.28 13.25)	11.63 (1.91 to 12.35)	0.63 (−0.50 to 1.70)	.25
ED/Obs^e^	8.53 (7.79 to 9.26)	9.64 (8.84 to 10.45)	−1.12 (−2.30 to 0.10)	.07	7.99 (7.24 8.74)	8.72 (8.30 to 9.14)	−0.73 (−1.90 to 0.40)	.21	8.6 (8.01 9.19)	9.44 (8.90 to 9.98)	−0.84 (−1.90 to 0.20)	.10

Abbreviations: ED, emergency department; Obs, observation; READI, Readiness Evaluation and Discharge Interventions Protocol.

^a^ Nurse assessment using nurse form of the Readiness for Hospital Discharge Scale (RHDS) with instruction to nurses to use their best judgment with the assessment information to guide actions in completing their patients’ preparation for discharge.

^b^ Patient self-assessment using the patient form of the RHDS followed by nurse assessment using the nurse form of the RHDS with instruction to nurses to use their best judgment with the assessment information to guide actions in completing their patients’ preparation for discharge.

^c^ Patient self-assessment using the patient form of the RHDS followed by nurse assessment using the nurse form of the RHDS with instruction to nurses to act and document nurse actions if the patient received a low readiness score (<7) on the nurse or patient form.

^d^ Adjusted for baseline and patient characteristics. Rates expressed per 100 index patient discharges. Adjusted rates and adjusted difference in rates were estimated using a multinomial logistic regression with adjustment for baseline event rates and patient characteristics and clustering at unit and hospital level.

^e^ We combined ED and observation as return to the hospital without inpatient readmission.

**Figure 2.**
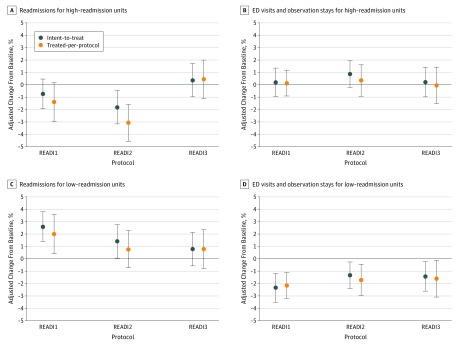
Analysis of Readiness Evaluation and Discharge Interventions (READI) Effectiveness for Intervention Units With Low (≤11.3%) and High (>11.3%) Readmission Rate at Baseline Difference-in-differences predictive margins with 95% CIs (error bars) of the absolute percentage point reduction from the READI intervention by protocol, estimated using a multinomial conditional likelihood difference-in-differences logistic model with adjustment for baseline event rates and patient characteristics with clustering at unit and hospital level. The READI1 protocol used the nurse form of the Readiness for Hospital Discharge Scale (RHDS) with instructions to nurses to use their best judgment with the assessment information to guide actions in completing their patients’ preparation for discharge. The READI2 protocol used patient self-assessment using the patient form of the RHDS followed by nurse assessment using the nurse form of the RHDS with instructions to nurses to use their best judgment. The READI3 protocol used patient self-assessment using the patient form of the RHDS followed by nurse assessment using the nurse form of the RHDS with instruction to nurses to act and document nurse actions if the patient received a low readiness score (<7) on the nurse or patient form. ED indicates emergency department.

The ITT analysis stratified for high and low baseline readmission rates revealed that, in high-readmission units, the adjusted readmission rate with READI2 decreased by 1.79 percentage points (95% CI, −3.20 to −0.40 percentage points; *P* = .009). We did not find an effect on ED/Obs visits with READI2 or on either outcome with READI1 and READI3. In TPP analysis, there was a significant reduction in readmission of 1.38 percentage points (95% CI, −2.50 to −0.30 percentage points; *P* = .02) with READI1, and the reduction in readmissions with READI2 was greater (3.05 percentage points; 95% CI, −4.50 to −1.60 percentage points; *P* < .001) (Figure 2; eTable 1 in Supplement 2).

On low-readmission units, the ITT effect was an increase in 30-day readmissions for READI1 (2.60 percentage points; 95% CI, 1.40-3.80 percentage points; *P* < .001) and for READI2 (1.41 percentage points; 95% CI, 0-2.80 percentage points; *P* = .05); there was no effect on readmissions with READI3. Concurrently, ED/Obs rates decreased with all protocols (READI1, −2.33 percentage points; 95% CI, −3.50 to −1.20 percentage points; *P* < .001 vs READI2, −1.32 percentage points; 95% CI, −2.40 to −0.20 percentage points; *P* = .02 vs READI3, −1.43 percentage points; 95% CI, −2.70 to −0.20 percentage points; *P* = .02). In TPP analyses, the results were similar with the exception that the increase in readmissions with READI2 was not significant (Figure 2; eTable 1 in Supplement 2).

### Sensitivity Analyses

In sensitivity analyses, the effects of the intervention were greater at 60 days for high-readmission units but did not extend beyond 30 days for low-readmission units (eTable 2 in Supplement 2). Excluding Saudi hospitals did not change the results (eTable 3 in Supplement 2). The intervention affected 30-day return to the hospital whether or not units had adopted a discharge transition program, although the effect was stronger in units with a program (eTables 4 and 5 in Supplement 2).

## Discussion

This cluster randomized clinical trial tested whether a unit-level intervention adding a structured discharge readiness assessment to usual care discharge practices reduces return to the hospital following discharge. The READI protocols were implemented within the context of variability in patient characteristics and disease conditions, clinicians who prepare patients for discharge, and units’ and hospitals’ usual care discharge practices and outcomes. In the full heterogeneous sample of hospitals, units, and patients, none of the READI protocols were consistently effective in reducing return to the hospital. However, the READI2 protocol, which included a structured discharge readiness assessment that incorporated the patient’s own perspective, significantly reduced rates of return to the hospital on units that had high readmission rates prior to implementation of the intervention.

The heterogeneous findings in high- vs low-readmission settings underscore the complexity of readmission reduction. Strategies for reducing readmission vary in effectiveness because of patient, clinician, and hospital factors.^4,5,6^ Our findings point to the broad utility of structured discharge readiness assessments in mitigating high rates of readmissions, but achieving readmission reduction in hospitals with already low rates is unlikely. On low-readmission units (≤11.3%), ED/Obs visits declined, but not readmissions. The finding of an increase in readmissions with the READI1 and READI2 protocols was unexpected, and the mechanism underlying this finding needs further exploration to determine and correct the cause. In considering structured discharge readiness assessment as a supplementary strategy to existing discharge practices, organizations with low readmission rates should evaluate their own potential for achieving further reduction in avoidable readmissions.

Incorporating the patient’s voice in clinical decision making about discharge preparation appears to be an important underlying mechanism for the effectiveness of structured discharge readiness assessment. In high-readmission units, the READI2 protocol more than doubled the absolute reduction in readmissions relative to the READI1 protocol that included only nurse assessment, with even greater additional reductions for TPP patients. On low-readmission units, protocols that included patient input on discharge readiness produced reduction in ED/Obs visits with small to nonsignificant concurrent increases in readmissions. Nurses on study units reported in focus groups that information provided in the patient self-assessment was valuable to opening conversations with patients about their unique discharge situations and needs. They also indicated that it provided them with documentation to support interprofessional conversations about discharge planning and timing.^29^ Improved patient-clinician and interprofessional communication is known to be positively associated with patient experience and overall quality of care.^30,31,32,33,34^

When adopting a structured discharge readiness assessment as a new patient care procedure, attention should be paid to consistent use of the protocol by nursing staff for all patient discharges. The READI intervention reached 70% of eligible patients and was more effective for these TPP patients. The improvement in effects of the intervention for TPP vs ITT patients suggests nonadherence to the discharge assessment protocol as a missed opportunity to improve outcomes.

Attention to informing, but not restricting, clinician judgment and to minimizing additional burden is an important consideration in planning implementation. The READI1 and READI2 protocols included an instruction to nurses to use their clinical judgment to determine appropriate actions in response to the discharge readiness assessments. An additional instruction during the READI3 protocol that prescribed scores that constitute low readiness and required documentation of nurse actions in response to low readiness scores may have inadvertently disincentivized more generalized attention to readmission risk-reduction efforts for all patients. We noted that RN-RHDS scores rose substantially with READI3, possibly indicating upcoding of assessment scores to avoid required documentation of actions for low readiness. The READI3 protocol was perceived by some nurses as burdensome within the busy workflow of the day of discharge, which may have interfered with intervention effectiveness.

Hospitals use a broad range of strategies for readmission avoidance that they customize to their organizational structure and culture. These strategies include, for example, use of case managers and discharge planning services, standardized education, transitional care programs, and pharmacy support.^3,6^ Overall, implementing a discharge readiness assessment program like the READI protocol is a low-intensity, low-cost intervention that can facilitate nurse-patient communication about discharge needs and stimulate communication with the care team. When used in conjunction with existing risk assessment tools for identifying high-risk patients, it provides a platform for assessing patients across the full range of risks for return to the hospital and identifying actionable needs for patients with low through high readmission risk.

### Limitations

This study had limitations. The sample was restricted to medical-surgical units from hospitals that had achieved Magnet designation for nursing excellence. The findings may differ in hospitals and units with different characteristics and levels of care quality.^35,36^ However, the hospitals and units were diverse by location, type, size, and baseline readmission rates; the READI intervention protocols were applied to a broad range of patients without targeting specific characteristics (advanced age or multiple chronic conditions) or diagnoses (cardiac, pulmonary, etc), allowing for testing in a heterogeneous sample of hospitals, units, and patients.

The only measure of fidelity to the READI protocols was whether the patient had an assessment completed (TTP patients). There was no monitoring of the actual processes of completion of the assessments, including when they were completed on the day of discharge. Treated-per-protocol patients were not selected at random, as selection was subject to nurse preferences and time constraints interacting with patient characteristics such as primary language, cognitive capacity, and sensory-motor skills needed for completing assessment scales. We adjusted for an extensive set of patient characteristics to minimize a treatment selection bias. Because this is an effectiveness trial of a unit-level intervention, the results reflect the outcomes that can be expected under actual clinical practice conditions.

Although efforts were made to minimize crossover effects between the intervention and control units, a small number of patients (<5%) in the final analysis sample were assessed by float nurses, some of whom may also have floated to the control unit. This may have led to understating the effectiveness of the intervention.

The analysis was limited to data for patients returning to the same hospital following discharge. However, the analysis method using same-hospital control units adjusted for factors related to out-of-hospital readmissions (hospital catchment area and system characteristics).

The results are subject to the limitations of data obtained from electronic records, including entry errors and missing data.^37^ The study team screened data on submission for outliers to expected ranges in each field and performed missing-at-random analysis and imputations.

## Conclusions

This multisite cluster randomized clinical trial demonstrated that adding a structured discharge readiness assessment that incorporates patients’ own perspective to usual discharge care practices holds promise for mitigating high rates of return to the hospital following discharge.

## Notes

Supplement 1. **Trial Protocol**

Supplement 2. **eAppendix.** Supplemental Methods **eReferences** **eTable 1.** Difference-in-Differences Estimates of the Effect of the READI Intervention on 30-Day Post-Discharge Return to Hospital: Full Model **eTable 2. **Difference-in-Differences Estimates of the Effect of the READI Intervention on 60-Day Post-Discharge Return to Hospital: Full Model **eTable 3.** Robustness of 30-Day Results, Without Saudi Hospitals (ITT n=137,814; TPP n=121,763) **eTable 4.** Robustness of 30-Day Results for Units With a Formal Discharge Model of Care in Place Prior to READI Study (ITT n=73,866; TPP n=53,187) **eTable 5.** Robustness of 30-Day Results for Units Without a Formal Discharge Model of Care in Place Prior to READI Study (ITT n=71,002; TPP n=56,252)

Supplement 3. **Data Sharing Statement**
