# Supplementary material for: Effect of Implementing Discharge Readiness Assessment in Adult Medical-Surgical Units on 30-Day Return to Hospital: The READI Randomized Clinical Trial
Source: JAMA Netw Open. 2019 Jan 25;2(1):e187387. doi: 10.1001/jamanetworkopen.2018.7387 (PMC6484543; doi:10.1001/jamanetworkopen.2018.7387)
Supplement: Supplement 1. — Trial Protocol [file jamanetwopen-2-e187387-s001.pdf]

READI (Readiness Evaluation And Discharge Interventions): Implementation as a Standard Nursing Practice for Hospital Discharge

STUDY PROTOCOL

STUDY RATIONALE

Executive Summary

Preparation of patients for discharge is a primary function of hospital-based nursing care and readiness for discharge is an important outcome of hospital care. Inadequacies in discharge preparation have been well-documented and linked to difficulty with self-management after hospital discharge and with increased likelihood of emergency department (ED) use and readmission. Prior studies by the research team have led to recommendations for implementation of discharge readiness assessment as a standard nursing practice for hospital discharge.

This study aims to evaluate the impact of unit-based implementation of discharge readiness assessment on readmission and ED use within 30 days post-discharge. Three protocols, each adding a component to discharge readiness assessment, will be used to introduce, in sequence: (1) discharge readiness assessment by the discharging nurse; (2) discharge readiness assessment by the discharging nurse informed by prior patient self-assessment of discharge readiness [patient-informed nurse assessment]; and (3) patient-informed nurse assessment, with the addition of an instruction to the discharging nurse to initiate and document nursing action(s) for patients with low readiness. Nurse and patient versions of the 8-item short form of the Readiness for Hospital Discharge Scale will be used for discharge readiness assessment.

The study will use a prospective, parallel cohort, stepped implementation design with four phase (baseline and the 3 discharge readiness protocols implemented in sequence) and two study conditions (implementation units and usual care control units). Difference-in-difference analysis will compare patient outcomes at baseline and each of the 3 phases on the implementation units (first difference) to outcomes on paired control units (second difference), adjusting for hospital, unit, and patient-level control variables. The optimal implementation protocol will be identified through these methods. The results will provide evidence of the impact of a hospital nursing care process on post-discharge outcomes, with important implications for patient well-being and ultimately costs of care. Process evaluation will assess implementation fidelity and context, facilitating broad translation as a standard of nursing practice for hospital discharge.

Background

Reducing readmission and ED utilization rates is central to health care improvement and reform efforts.[1] Recent research has linked hospital nurse staffing to readmission rates. [2-3] With Medicare readmission rates approaching 20% [4] and financial penalties for high rates of 30 day readmission, novel approaches to engaging hospital nurses in readmission reduction efforts hold significant promise for promoting high-quality affordable patient care.

Problems with hospital discharge are well documented. Perceived inadequacies in discharge planning, teaching, and coordination are associated with greater likelihood of post-discharge problems, ED use, and readmission.[5-9] Most readmissions within 30 days are viewed as preventable [10] and failures of discharge preparation.[11]

Large scale initiatives to improve discharge transitions have focused on communication and coordination of care between hospital and community providers using specialized roles for transition support.[8,12-15] The role of the acute care staff nurse has been virtually ignored in discharge transition initiatives, despite the fact that, in most hospitals, the staff nurse is responsible for the complex processes of preparing patients for discharge.[16-17]

Readiness for discharge is an outcome of discharge preparation. In previous research by this study team, the 21-item Readiness for Hospital Discharge Scale (**RHDS**) has been developed and tested with multiple inpatient groups. [18-22] In measuring readiness for discharge to home following adult medical-surgical hospitalization, patient self-assessment [**PT-RHDS**] and nurse assessment [**RN-RHDS**] forms of the scale have demonstrated an association with post-discharge utilization (readmissions and ED visits).[3,19,23] The RHDS was reduced to an 8-item version for use in clinical practice. The 8-item RN-RHDS was more strongly associated with readmission than the 8-

item PT-RHDS in 2 adult samples in the Midwest and Eastern US.[23-24] Patients with low readiness by nurse assessment experienced more than a 6-fold increase in the likelihood of being readmitted.[24]

Currently there is no standard approach or tool available for routine use in clinical practice. The body of evidence from prior studies by the research team lays the foundation for the recommendation that pre-discharge readiness assessment be implemented as a standard nursing practice for hospital discharge.[3]

Aims

We propose to conduct a multi-site study to determine the impact on post-discharge utilization (readmission and ED visits) and costs of implementing discharge readiness assessment as a standard nursing practice for adult medical-surgical patients discharged to home, using a reliable, valid, and clinically meaningful tool (RHDS 8-item short form). The value of implementing discharge readiness assessment as a standard practice lies in timely, rapid, and systematic determination of nurse and patient perspectives on 4 aspects of discharge readiness (personal status, knowledge, coping ability, expected support). The assessment results enable the nurse to initiate pre-discharge risk-mitigating actions that enhance patient readiness and avert adverse post-discharge outcomes that may result in ED visits and readmissions.

The proposed research extends the research team's prior observational studies to an implementation study. The study tests, in a stepped approach, the impact of implementing discharge readiness assessment by the discharging nurse as standard nursing practice, and the incremental value of informing the nurse assessment with the patient's perspective, and of requiring that the nurse initiates and documents risk-mitigating actions for patients with low readiness scores.

Specific Aim 1: Determine if discharge readiness assessment by the discharging nurse using the RN-RHDS-short form (RN-RHDS protocol), when implemented as a standard pre-discharge nursing practice, contributes to reduced readmissions and ED visits within 30 days post-discharge.

Specific Aim 2: Determine if nurse assessment informed by patient self-assessment using the PT-RHDS short form contributes to improved post-discharge outcomes (readmission and ED use within 30 days post-discharge) by adding patient's perspective to the RN-RHDS protocol (RN-RHDS+PT-RHDS protocol).

Specific Aim 3: Determine if adding a structured format for documenting nurse actions triggered by low discharge readiness assessment scores improves patient outcomes (readmission and ED use within 30 days post-discharge).

RESEARCH DESIGN

A prospective, parallel cohort, stepped implementation study design [25] with difference-in-difference analysis will be used to evaluate outcomes (likelihood of readmissions and ED visits within 30 days post-discharge) for 4 sequential cohorts of patients on units where usual care (baseline cohort 0) is followed by a stepwise implementation of 3 discharge readiness assessment protocols (cohort 1[**AIM1**]:RN-RHDS protocol; cohort 2 [**AIM2**]: RN-RHDS+PT-RHDS protocol; cohort 3 [**AIM3**]: RN-RHDS+PT-RHDS+NDAG protocol), compared to 4 concurrent cohorts of patients on usual care/control units. Patient outcome (readmission/ED use) differences are examined between each sequential implementation cohort (first difference) while also controlling for any changes in institutional practices or seasonal trends using usual care cohorts at each concurrent phase (second difference). The implementation will occur at the unit-level, incorporating the implementation protocols into unit operational processes for hospital discharge. The decision to implement at the unit-level, with comparable units serving as control units, was made because spill-over effects of nurse training and cross assignment of nurses to implementation and control patients on the same unit would likely contaminate the control patient samples.

We will use the following timeline for data collection on each unit.

eTable 1

Phases / Study Units	Baseline<br>4 months	READI1<br>4 months	READI2<br>4 months	READI3<br>4 months

Implementation	Baseline	RN-RHDS	RN-RHDS<br>+PT-RHDS	RN-RHDS<br>+PT-RHDS +NDAG
Control	Concurrent control	Concurrent control	Concurrent control	Concurrent control

Sample

This study will use a multi-level nested sample consisting of patients, cared for by nurses, within units, within hospitals. The patient sample will include adult patients (18+) being discharged home with or without home care services from medical-surgical nursing units of 40 Magnet-designated hospitals we plan to enroll. On the implementation units, all eligible patients and their discharging nurses will be included. On control units, all eligible patients will be included following the same sample inclusion criteria. All nursing staff performing the final discharge preparation on the day of discharge, including RNs and Licensed Practical Nurses, will be included in the study.

We will include two units per participating hospital. One will be randomly selected as the implementation unit and the other as the control unit. Where possible, these units will be of s similar type (either medical, surgical, or medical-surgical). Critical care units and oncology specialty units will be excluded. We will work with hospitals to achieve a balance of medical, surgical, and mixed medical-surgical unit types across the hospitals.

Sample Size and Power Analysis: For the study model with a full set of stand-alone and interaction terms for a 4x2 difference-in-difference design (4 study phases [0/1/2/3] and 2 study conditions [implementation/usual care]) with control variables (patient characteristics, unit-level nurse staffing controls, hospital fixed effects), the minimum sample size required to achieve 80% power and p<.05 significance for small effect sizes (.02 change in R^2) in main study aims, including subgroup analysis for patients with low readiness (Aim 3) is 24,304 patients.[26] For 40 hospitals, the sample would be 301 patients per implementation and control unit, with 76 patients per unit in each of the 4 phases. To account for clustering at the 4x2 cohort-implementation phase cells, the data will be oversampled[26] by 50% for the final sample of 452 per unit (113 per unit per phase). Assuming a range of 75 to 200 discharges per unit per month and 50% of patients meeting inclusion criteria for discharge to home (55% in our prior study), we expect to accrue the target sample on smaller study units in 4 months. To ensure that the results of the analyses can be properly adjusted for confounding due to any unrelated system-wide changes in patient care practices or seasonal trends that may occur during the study period, the start and end date of each implementation and data collection phase will be same for paired implementation and control units; frequency weighting will be used to adjust for sample size differences among units.

Because the study design includes a time trend, patients will be enrolled throughout the entire study period as described in the study design. Therefore, the number of patients enrolled per hospital will vary based on volume and will exceed the minimum sample estimates.

INTERVENTION PROTOCOLS

The study will evaluate implementation of assessment of readiness for discharge for adult medical-surgical patients being discharged to home. We use a stepped implementation approach with sequential introduction of structured tools for discharge readiness assessment and documentation of nursing actions to improve discharge transition outcomes for patients with low readiness. We will evaluate the incremental benefits of 3 protocols: (1) RN-RHDS; (2) RN-RHDS+PT-RHDS; (3) RN-RHDS+PT-RHDS+NDAG.

<u>**READI1** protocol</u>: The **RN-RHDS/SF (Nurse assessment of Readiness for Hospital Discharge Scale/short form)** will be used for the nurse assessment of patient's readiness for discharge. The RN-RHDS/SF consists of 8 items from a longer 21 item scale that both use a 0-10 scaling format, with higher scores indicating greater readiness. The 21-item instrument has reliability estimates in adult medical-surgical patients age 18 to 102 of >.80

for total and subscales and confirmatory factor analyses in 2 studies have supported a 4-factor structure (personal status, knowledge, perceived coping ability, expected support).[3,19] RN-RHDS/SF is a reduced form of the instrument that retains 2 items per factor; Cronbach's alpha reliability estimates exceed .80. RN-RHDS/SF (explaining 93% of long form variance) was associated with a 6-9 fold increase in odds of readmission in models unadjusted and adjusted for numerous patient characteristics. [24]

For the READI1 protocol, the discharging nurse was instructed to complete the form on the day of hospital discharge normally within 4 hours prior to discharge, normally after completion of discharge preparation.

<u>READI2</u> <u>protocol</u>: The **PT-RHDS/SF** (Patient Readiness for Hospital Discharge Scale/short form) is a parallel version of the RN-RHDS form that is completed by patient self-assessment. PT-RHDS was inversely associated post-discharge coping difficulty,[19] readmission within 30 days following hospital discharge[19] and ED visits.[3] The positive predictive value of PT-RHDS for readmission progressively increased with age, especially in the oldest (85 years and older).[27]

For the READI2 protocol, the patient completed the PT-RHDS and the responses were reviewed by the discharging nurse to inform the nurse of the patient's perspective. The nurse then completed the RN-RHDS form to document the nurse's assessment that considered all relevant information available to the nurse.

<u>READI3</u> <u>protocol</u>: The NDAG (Nurse Discharge Action Guide) consists of a list of potential nursing actions that can be initiated related to discharge transition developed from literature review. The NDAG was completed by the discharging nurse after the PT-RHDS and RN-RHDS were completed. Nurses will be instructed that initiation and documentation of an action on the NDAG was required for any patient with a score of <7 on any item of the RN-RHDS or PT-RHDS. The cut-off score of <7 was derived from a prior study.[24]

The **RN-RHDS short form** will be used for the nurse assessment of patient's readiness for discharge. A parallel patient form, the **PT-RHDS short form**, will be used for patient self-assessment. The RN-RHDS/PT-RHDS are 8-item short forms of a 21-item instrument that has undergone rigorous testing. The 21-item instrument has reliability estimates in adult medical-surgical patients age 18 to 102 for both RN-RHDS and PT-RHDS of >.80 for total and subscales and confirmatory factor analyses in 2 studies have supported a 4-factor structure (personal status, knowledge, perceived coping ability, expected support).[3,19] Using the long forms of the instrument, PT-RHDS was inversely associated with readmission within 30 days following hospital discharge[19] and ED visits.[3] The positive predictive value of PT-RHDS for readmission progressively increased with age, especially in the oldest (85 years and older).[27] In a subset (n=162) of the 1892 adult medical surgical patients in the Weiss et al.[3] study, RN-RHDS was associated with post-discharge utilization (readmission or ED visit) with an odds ratio of .57 indicating a 43% reduction in utilization for a 1 point increase in the RN-RHDS (on a 10 point scale), while PT-RHDS showed no association.[23]

Item reduction resulted in a short form with 8 items (2 per subscale) that explain 94% of RN-RHDS and 93% of PT-RHDS scale variance.[24] In its reduced forms, Cronbach's alpha reliability estimates for RN-RHDS and PT-RHDS were .82 and .83 using the dataset from Weiss et al.[3] In a replication of the Weiss et al.,[27] study with 254 adult medical-surgical patients and their discharging nurses using the RHDS short forms in place of the long forms, RN-RHDS again was highly predictive of 30-day readmission and the nurse form was more predictive than the patient form. Using a cutoff score of <7 as indicative of low readiness, RN-RHDS short form was associated with a 6-9 fold increase in odds of readmission even in the model adjusted for numerous patient characteristics.[24]

The NDAG consists of a list of potential nursing actions that can be initiated related to discharge transition support. The NDAG (see Appendix) consists of 2 columns: actions initiated prior to discharge readiness assessment and those initiated in response to discharge readiness assessment. The NDAG was developed from literature review and has undergone pilot testing in a sample of 44 patients. Results indicated that 45% of patients had nursing actions initiated, with one quarter of these being triggered after the completion of the RHDS assessment. Ten percent (n=4) reported low readiness scores; all had actions prior to the assessment and one had an additional action initiated after the assessment. The findings of this pilot study (manuscript in preparation) suggest that nurses intervene for readiness risk both in anticipation of discharge and at the time of discharge readiness assessment and that the use of

the NDAG can serve as a trigger for action by the nurse. We will analyze total nurse actions as well as differentiate between anticipatory and reactive nurse actions.

For this study, the NDAG will be completed following discharge readiness assessment (RN-RHDS+PT-RHDS) for all patients on the implementation units. Nurses will be instructed that initiation and documentation of an action on the NDAG is essential for any patient with a score of <7 on any item of the RN-RHDS or PT-RHDS. To facilitate completeness of data capture, nurses will be provided with an open-ended response option that will be coded for content by the researchers. The open-ended response will capture any actions taken by the nurse that are not included in the list of actions on the NDAG.

MEASURES AND OUTCOMES

Independent Variables: For this difference-in-difference study design, the independent variables are a set of indicators each corresponding to one of the 8 possible combinations of being hospitalized on an implementation or a control unit during one of the 4 study phases. **Dependent variables (Outcomes of implementation):** The measured outcomes of the implementation will be readmissions and ED visits in the first 30 days post-discharge. Data for these outcomes will be extracted from hospital electronic information systems. **Control variables:** The following **patient characteristics** will be extracted from electronic hospital records: age, sex, race/ethnicity, APR-DRG with severity and mortality indices, and type of admission (medical/surgical), discharge disposition (home, home with home health), length of stay, ICU stay, and payer. Based on our prior research that demonstrated a relationship between RN hours per patient day (RNHPPD) and readmissions,[3] we will include the following **unit-level nurse staffing variables** in the analytic models: RNHPPD, skill mix (% RN, %BSN). These data will be collected monthly from nursing administrative databases and specifications will be consistent with NQF/NDNQI.

STUDY SITE EDUCATION AND TRAINING PROCEDURES

Education regarding the implementation protocols and training in study procedures will occur at multiple levels and times throughout the study. A site Principal Investigator will serve as the master trainer and coordinator of training for unit nurses. Site PIs/Master Trainers will be trained by the study team. The training will consist of a detailed review of study design and procedures, and of methods for training staff nurses. At each phase, we will include an educational component that introduces nurses to key informational content to support effective use of the tool being introduced. In phase 1, the content will include information on the evidence base related to nurse assessment of discharge readiness and a detailed overview of the RN-RHDS. In phase 2, the content will include evidence about the inclusion of patient voice in nursing assessment to improve patient experience of care and outcomes and a detailed overview of the PT-RHDS. In phase 3, the content will include importance of assessment of low readiness as a trigger for nursing action, information about specific nursing actions to mitigate the potential adverse outcomes associated with low readiness, and advocacy for follow-up care services for patients with low readiness.

Site PI training will occur via webinar/videoconferencing using Go-To-Meeting®. All training documents and all study materials will be available on the study website. Start-up training for unit nurses will be conducted by site PIs in the first 2 weeks of phase 1, followed immediately by the initiation of data collection. Sites will commit up to 2 hours of training per unit staff member for the startup training. The site PI will use video presentations prepared by the study team and PowerPoint slides for staff training on (1) the purpose of the study and the evidence base for discharge readiness assessment, and (2) a detailed review of the RN-RHDS instrument and study procedures, including human subjects' protection. All training materials will be available to sites on the designated website. PowerPoint slides will contain voice-over for online training purposes for staff not able to attend in-person training. Training for phases 2 and 3 will be via in-person or online training, as selected by site PIs.

Because this is a unit-based implementation approach, logs will be kept of participation in training to assure all staff nurses have been trained. For staff not attending, site PIs will assure that training has occurred by documentation of review of study materials posted on the study website. Staff will login to the site to confirm that they have met the training requirement.

HUMAN SUBJECTS PROTECTION

Hospital IRBs: The study protocol will be reviewed and approved by the researchers' university IRBs. Each local IRB will also review and make a determination.

Human Subjects Protection: The study **evaluates the implementation of** a process of care that augments existing discharge processes. The contribution of the acute care clinical nurse in the complex processes of preparing patients for discharge is a study focus. Implementation of a new protocol for nurse assessment of a patient's readiness for discharge will occur in 3 sequential phases to evaluate each of 3 components of the discharge readiness assessment protocol (1. Nurse assessment; 2. Nurse assessment informed by patient self-assessment [patient-informed nurse assessment]; 3. Patient informed nurse assessment plus an instruction to identify appropriate interventions if discharge readiness assessment indicated low readiness). Implementation of the protocol in sequence will be conducted on entire nursing units (unit –level implementation) with all nurses trained in the protocol and used with all patients discharged to home. Outcomes on implementation units will be compared with paired usual care (control) units. Outcomes of interest are readmissions and ED visits post-discharge.

The READI study received expedited approval from Marquette University's IRB on September 9, 2013. Marquette University IRB will serve as the primary IRB for the study. All participating hospital IRBs will review and make a determination of the most appropriate mechanism for IRB approval. We encourage the execution of an Institutional Authorization Agreement with Marquette University IRB to facilitate continuing review during the 3-year study period.

We provide the following information to consider in making a determination of the appropriate mechanism for IRB approval at hospital sites:

We are requesting **a waiver of documentation of consent** based on the following factors:

1. The implementation is at the unit level and involves standardization of an assessment that is often informally conducted by nurses in the course of their practice and is consistent with professional standards for nursing care.
2. There is expected benefit to patient outcomes from implementing the discharge readiness assessment and if successful will reduce risk of readmission. In addition, there are no identifiable risks associated with the implementation protocols.
3. With unit-based implementation, the data cannot be reasonably collected without the waiver.
4. Patient self-assessment of discharge readiness [PT-RHDS] is used to inform the nurse assessment of discharge readiness and is integrated into discharge-related clinical care processes. All other patient data are extracted from electronic medical records. There is no direct contact with control units.
5. The PT-RHDS contains an introductory statement indicating the information is being used to determine the benefits of a new way for the patient's nurse to evaluate readiness for discharge.
6. The data will be de-identified by the site PI before release to the research team.

All data will be **de-identified at the study site by the site PI**. Nurse RHDS Assessment forms will contain a section at the bottom of the patient study ID and the patients label containing identifying information. After completion by the discharging nurse, these forms will be collected by the site PI, the identification section will be detached from the form. The de-identified RHDS forms will be sent to the study team; the detached identifiers with link to study ID will be used by the site PI to create a study log on an excel spreadsheet which will be retained at the study site. The study log will be password protected by the site PI. This log will be given to IT services to generate the electronic data files for the study. The site PI will verify completeness of the electronic extract and then remove all identifiers from the file. Following verification of transmission to the study team via secure FTP and screening of the transmitted file, the site PI will destroy all on-site paper and electronic identifiers. The site PI will retain a copy of the de-identified electronic file.

Data Protection: All data will be de-identified at the study site. Nurse RHDS Assessment forms will contain a section at the bottom of the patient study ID and the patients label containing identifying information. After completion by the discharging nurse, these forms will be collected by the site PI, the identification section will be detached from the form. The de-identified RHDS forms will be sent to the study team; the detached identifiers with

link to study ID will be used to create a study log on an excel spreadsheet which will be retained at the study site. The study log will be password protected by the site PI. This log will be given to IT services to generate the electronic data files for the study.

The site PI will verify completeness of the electronic extract and then remove all identifiers from the file. Following verification of transmission to the study team via secure FTP and screening of the transmitted file, the site PI will destroy all on-site paper and electronic identifiers. The site PI will retain a copy of the de-identified electronic file.

Data will be submitted to the primary study site at Marquette University. Data will be uploaded by study sites to a secure Office 365 password protected database. Site will have access only to their own secure space and will not be able to access data submitted by other participating hospitals.

References

1. Naylor MD, Aiken LH, Kurtzman ET, Olds DM, Hirschman KB. The care span: The importance of transitional care in achieving health reform. *Health Affairs*. 2011;30(4):746-754.
2. McHugh MD, Ma C. Hospital nursing and 30-day readmissions among Medicare patients with heart failure, acute myocardial infarction, and pneumonia. *Med Care.* 2013;51(1):52-59.
3. Weiss M, Yakusheva O, Bobay, K. (2011). Nursing staffing, readiness for hospital discharge, and post-discharge utilization. *Health Serv Res.* 2011;46(5):1473-1494.
4. Jencks SF, Williams MV, Coleman EA. Rehospitalizations among patients in the Medicare fee-for-service program. *New Engl J Med*. 2009;360(14):1418-1428.
5. Banja J, Eig J, Williams M. Discharge dilemmas as system failures. *Am J Bioethics.* 2007;(3),29-31.
6. Bull MJ, Hansen HE, Gross CR. A professional-patient partnership model of discharge planning with elders. *Appl Nurs Res. 2000;13*(1):18-28.
7. Coleman EA, Mahoney E, Parry C. Assessing the quality of preparation for posthospital care from the patient's perspective: the care transitions measure. *Med Care.* 2005;43:246–255.
8. Henderson A, Zernike W. A study of the impact of discharge information for surgical patients. *J Adv Nurs.* 2001;35(3):435-41.
9. Jack BW, Chetty V K, Anthony D. et al. A reengineered hospital discharge program to decrease rehospitalization: A randomized trial. *Ann Intern Med.* 2009;150(3):178-187.
10. Medicare Payment Advisory Commission [Medpac]. *Report to the congress: Promoting greater efficiency in Medicare*. Washington, DC: Medicare Payment Advisory Commission; 2007.
11. Minott, J. (2008). *Reducing hospital readmissions*. Washington, DC: AcademyHealth. http://www.academyhealth.org/files/publications/Reducing_Hospital_Readmissions.pdf**.** Accessed September 24, 2018.
12. Coleman EA, Parry C, Chalmers SA, Min S-J. The care transition intervention: Results of a randomized clinical trial. *Arch Intern Med.* 2006;166*:*1822-1828.
13. Naylor MD, Brooten D, Campbell R, Jacobsen BS, Mezey MD, Pauly MV. Comprehensive discharge planning and home follow-up of hospitalized elders: A randomized clinical trial. *J Amer Med Assoc.* 1999;281(7),613-620.
14. Naylor MD, Brooten DA, Campbell RL, Maislin G, McCauley KM, Schwartz JS. Transitional care of older adults hospitalized with heart failure: a randomized, controlled trial. *J Am Geriatr Soc.* 2004;52(5):675–84.
15. Nielsen GA, Bartely A, Coleman E, et al. *Transforming care at the bedside how-to guide: Creating and ideal transition home for patients with heart failure*. Cambridge, MA: IHI; 2008. http://www.ihi.org. Accessed September 24, 2018.
16. Foust JB. Discharge planning as part of daily nursing practice. *Appl Nurs Res.* 2007;20(2):72-77.
17. Nosbusch JM, Weiss ME, Bobay KL. An integrated review of the literature on challenges confronting the acute care staff nurse in discharge planning. *J Clin Nurs.* 2011;20(5-6):754-774.
18. Weiss ME, Piacentine LB. Psychometric properties of the Readiness for Hospital Discharge Scale. *J Nurs Measurement.* 2006;14(3):163-180.
19. Weiss ME, Piacentine LB, Lokken L, et al. (2007). Perceived readiness for hospital discharge in adult medical-surgical patients. *Clin Nurse Spec.* 2007;21:31-42.
20. Weiss M, Johnson N, Malin S, Jerofke T, Lang C, Sherburne E. Readiness for discharge in parents of hospitalized children. *J Pediatr Nurs.* 2008;23(4),282-295.
21. Weiss M, Lokken L. Predictors and outcomes of postpartum mothers' perceptions of readiness for discharge after birth. *J Obstet Gynecol Neonatl Nurs.* 2009;38(4):406-417.
22. Lerret S, Weiss ME. How ready are they? Parents of pediatric solid organ transplant recipients and the transition from hospital to home following transplant. *Pediatr Transpl.* 2011;15:606–616.
23. Weiss,ME, Yakusheva O, Bobay KL. Nurse and patient perceptions of discharge readiness in relation to postdischarge utilization. *Med Care.* 2010;48(5):482-486.
24. Weiss ME, Costa L, Yakusheva O, Bobay K. Validation of patient and nurse short forms of the Readiness for Hospital Discharge Scale and their relationship to return to the hospital. *Health Serv Res.* 2014;49(1):304-317.

25. Handley MA, Schillinger D, Shiboski S. Quasi-experimental designs in practice-based research settings: Design and implementation considerations. *J Am Board Fam Med.* 2011;24:589–596.
26. Ender PB. STATA: Power logistic regression (powerlog) *Stata Statistical Software: Release 12*. College Station, TX: StataCorp LP; 2011.
27. Bobay K, Jerofke T, Weiss M, Yakusheva O. Age-related differences in perception of quality of discharge teaching and readiness for hospital discharge. *Geriatr Nurs. 2010;31(3):178-187*.

READINESS FOR HOSPITAL DISCHARGE SCALE -- ADULT FORM ©

Please fill in the circle next to your answer. The answers are on a 10-point scale from 0 to 10. The words below the number indicate what the 0 or the 10 means. Pick the number between 0 and 10 that best describes how you feel. For example, circling number 7 means you feel more like the description of number 10 than number 0 but not completely.

1. How physically **ready** are you to go home?	1 2 3 4 5 6 7 8 9 10<br>Not ready ... Totally ready
2. How would you describe your **energy** today?	1 2 3 4 5 6 7 8 9 10<br>Low energy ... High energy
3. How much do you **know about problems to watch for** after you go home?	1 2 3 4 5 6 7 8 9 10<br>Know nothing at all ... Know all
4. How much do you **know about restrictions (**what you are allowed and not allowed to do) after you go home?	1 2 3 4 5 6 7 8 9 10<br>Know nothing at all ... Know all
5. How well will you be able to **handle the demands** of life at home?	1 2 3 4 5 6 7 8 9 10<br>Not at all ... Extremely well
6. How well will you be able to **perform your personal care** (for example, hygiene, bathing, toileting, eating) at home?	1 2 3 4 5 6 7 8 9 10<br>Not at all ... Extremely well
7. How much **help** will you have if needed with your **personal care** after you go home?	1 2 3 4 5 6 7 8 9 10<br>None ... A great deal
8. How much **help** will you have if needed with your **medical care** needs (treatments, medications) after you go home?	1 2 3 4 5 6 7 8 9 10<br>None ... A great deal

© Dr. Marianne Weiss, DNSc, RN
marianne.weiss@marquette.edu

READINESS FOR HOSPITAL DISCHARGE SCALE – ADULT - NURSE SHORT FORM ©

You are being asked to assess the readiness for discharge of your hospitalized patient. Please complete the form within the 4 hours before the patient leaves your unit.

Please fill in the circle next to your answer. The answers are on a 10-point scale from 0 to 10. The words below the number indicate what the 0 or the 10 means. Pick the number between 0 and 10 that best describes how you feel. For example, circling number 7 means you feel more like the description of number 10 than number 0 but not completely.

1. How physically **ready** is your patient to go home?	1 2 3 4 5 6 7 8 9 10<br>Not ready Totally ready
2. How would you describe your patient's **energy** today?	1 2 3 4 5 6 7 8 9 10<br>Low energy High energy
3. How much does your patient **know about problems to watch for** after going home?	1 2 3 4 5 6 7 8 9 10<br>Know nothing at all Know all
4. How much does your patient **know about restrictions (**what he/she is allowed and not allowed to do) after going home?	1 2 3 4 5 6 7 8 9 10<br>Know nothing at all Know all
5. How well will your patient be able to **handle the demands** of life at home?	1 2 3 4 5 6 7 8 9 10<br>Not at all Extremely well
6. How well will your patient be able to **perform his/her personal care** (for example, hygiene, bathing, toileting, eating) at home?	1 2 3 4 5 6 7 8 9 10<br>Not at all Extremely well
7. How much **help** will your patient have if needed with his/her **personal care** after going home?	1 2 3 4 5 6 7 8 9 10<br>None A great deal
8. How much **help** will your patient have if needed with his/her **medical care** needs (treatments, medications)?	1 2 3 4 5 6 7 8 9 10<br>None A great deal

© Dr. Marianne Weiss, DNSc, RN
marianne.weiss@marquette.edu
