# Supplementary material for: Effect of Implementing Discharge Readiness Assessment in Adult Medical-Surgical Units on 30-Day Return to Hospital: The READI Randomized Clinical Trial
Source: JAMA Netw Open. 2019 Jan 25;2(1):e187387. doi: 10.1001/jamanetworkopen.2018.7387 (PMC6484543; doi:10.1001/jamanetworkopen.2018.7387)
Supplement: Supplement 2. — eAppendix. Supplemental Methods eReferences eTable 1. Difference-in-Differences Estimates of the Effect of the READI Intervention on 30-Day Post-Discharge Return to Hospital: Full Model eTable 2. Difference-in-Differences Estimates of the Effect of the READI Intervention on 60-Day Post-Discharge Return to Hospital: Full Model eTable 3. Robustness of 30-Day Results, Without Saudi Hospitals (ITT n=137,814; TPP n=121,763) eTable 4. Robustness of 30-Day Results for Units With a Formal Discharge Model of Care in Place Prior to READI Study (ITT n=73,866; TPP n=53,187) eTable 5. Robustness of 30-Day Results for Units Without a Formal Discharge Model of Care in Place Prior to READI Study (ITT n=71,002; TPP n=56,252) [file jamanetwopen-2-e187387-s002.pdf]

Supplementary Online Content

Weiss ME, Yakusheva O, Bobay KL, et al; READI Site Investigators. Effect of implementing discharge readiness assessment in adult medical-surgical units on 30-day return to hospital: the READI randomized clinical trial. *JAMA Netw Open*. 2019;2(1):e187387. doi:10.1001/jamanetworkopen.2018.7387

eAppendix. Supplemental Methods

eReferences

eTable 1. Difference-in-Differences Estimates of the Effect of the READI Intervention on 30-Day Post-Discharge Return to Hospital: Full Model

eTable 2. Difference-in-Differences Estimates of the Effect of the READI Intervention on 60-Day Post-Discharge Return to Hospital: Full Model

eTable 3. Robustness of 30-Day Results, Without Saudi Hospitals (ITT n=137,814; TPP n=121,763)

eTable 4. Robustness of 30-Day Results for Units With a Formal Discharge Model of Care in Place Prior to READI Study (ITT n=73,866; TPP n=53,187)

eTable 5. Robustness of 30-Day Results for Units Without a Formal Discharge Model of Care in Place Prior to READI Study (ITT n=71,002; TPP n=56,252)

This supplementary material has been provided by the authors to give readers additional information about their work.

© 2019 Weiss ME et al. *JAMA Network Open.*

eAppendix: Supplemental Methods

In this supplement, we will first demonstrate the difference-in-differences approach using a hypothetical numerical example, and then we will show how this approach was applied in our study.

A difference-in-differences analysis[1-2] is applicable when a policy or an intervention is evaluated using a pre-/post-comparison method when other confounding trends could potentially affect the outcome of interest. For example, in evaluating whether an introduction of a new readmission reduction initiative in an inpatient unit successfully reduces readmissions, a difference-in-differences analysis will remove concurrent hospital-level readmission reduction efforts, hospital-level quality improvement efforts, and seasonal changes when estimating the effectiveness of the unit-level intervention. In multi-site studies, national policy and spurious global trends can also play a role. Not properly accounting for these contemporaneous trends could lead to spurious results, such as misattributing an observed readmission reduction from an unrelated hospital-level initiative to the unit-level intervention of interest or, alternatively as, misinterpreting globally rising readmission rates as an ineffective intervention.

Intuitively, a difference-in-differences analysis decomposes the observed changes in readmissions into two “differences”. First, it computes changes in the observed outcome from baseline for both the intervention and the control setting (first difference), and then it subtracts the calculated changes in the observed outcome on the control unit (second difference). The hypothetical example below demonstrates how difference-in-differences analysis works:

© 2019 Weiss ME et al. *JAMA Network Open.*

Hypothetical Example of the Difference-in-differences approach		
	**Pre-intervention (baseline)**	**Post-intervention**
	Observed outcome data (per 100 discharges)	
**Control unit**	10	9
**Intervention unit**	8	6
	First difference	
**Control unit**	[ref]	-1
**Intervention unit**	[ref]	-2
	Second difference (difference-in-differences)	
**Control unit**		
**Intervention unit**	[ref]	-1

When the outcomes data are available at the patient level instead of aggregated at the unit level, a difference-in-differences regression model can be used to obtain the difference-in-differences effect estimates. The regression approach is stronger than a simple unit-level difference-in-differences calculation because it adjusts for patient characteristics directly, instead of assuming that they were the stable across time (pre- and post-intervention) and study conditions (intervention and control). To execute this approach, two new indicator variables are created: one for the Time period (T) for pre- versus post-intervention and one for the Study Condition (SC) for intervention versus control. All patients discharged during the post-intervention period are assigned the value of "1" for the T variable, while all patients discharged during the pre-intervention period are assigned a "0" and represent the reference category for the first difference. Similarly, all patients discharged post-intervention implementation are assigned the value of "1" for the SC variable, with those discharged from the control unit assigned a "0" as reference for the second difference.

		**TIME PERIOD (T)**	
		**Pre-intervention measurement/ "0"**	**Post-intervention measurement/ "1"**
**STUDY CONDITION (SC)**	**Control unit/ "0"**	T = 0<br>SC = 0	T = 1<br>SC = 0
	**Intervention unit/ "1"**	T = 0<br>SC = 1	T = 1<br>SC = 1

© 2019 Weiss ME et al. *JAMA Network Open.*

Using "y" to represent the outcome (e.g., readmissions), the difference-in-differences regression is set up as following:

$$Logit\ P(y=1) = regression\ intercept + \beta_1 (T) + \beta_2 (SC) + \beta_3 [(T)\times(SC)] + X'b \qquad [1]$$

where P(y=1) is the probability that a patient is readmitted, and [(T) ×(SC)] is the interaction term, or the scalar product of the T and SC indicator variables, and X's are patient control variables times their regression coefficients b's, written in a vector notation. This model is estimated with data grouping at the unit level using a standard logistic regression approach.

In regression model [1], the change in the log-transformed probability of readmission P(y) from the pre-intervention baseline (T=0) to post-intervention (T=1) is measured by $\beta_1 + \beta_3$ (SC), and it takes two values depending on the value of SC. For the control unit (SC=0), the change in the probability of a readmission is estimated by β_1, and it reflects any readmission trend unrelated to the intervention of interest. For the intervention unit, (SC=1) the change in the log-transformed probability of readmission is estimated by $\beta_1 + \beta_3$, which can be decomposed into a change due to the unrelated trends, β_1, and a change specific to the intervention unit due to the implementation of the intervention, β_3. Therefore, β_3 is a difference-in-differences estimate for which this approach allows one to obtain uncertainty bounds and carry out hypothesis testing as with any other conventional regression coefficient. Note that the difference-in-differences estimate β_3 is baseline-adjusted and it is also fully adjusted for patient characteristics.

Applied to testing the effectiveness of the READI intervention, we created three time period indicator variables, T1, T2, and T3, for the three respective sequentially implemented protocols, and a study condition variable SC equal to 1 for patients discharge from the 33 intervention units (0 for patients discharged from the 33 control units). Note that each record has three time period variables, regardless of the record's time period. For instance, an intervention unit record collected during time period 2 will have the values T1 = 0, T2 = 1, T3 = 0, and SC = 1. The below figure illustrates how each indicator would be assigned during each period.

		Pre-intervention	READI Protocol 1	READI Protocol 2	READI Protocol 3
**Control unit**	**T1**	T1 = 0	T1 = 1	T1 = 0	T1 = 0
	**T2**	T2 = 0	T2 = 0	T2 = 1	T2 = 0
	**T3**	T3 = 0	T3 = 0	T3 = 0	T3 = 1
	**SC**	SC = 0	SC = 0	SC = 0	SC=0
**Intervention unit**	**T1**	T1 = 0	T1 = 1	T1 = 0	T1 = 0
	**T2**	T2 = 0	T2 = 0	T2 = 1	T2 = 0
	**T3**	T3 = 0	T3 = 0	T3 = 0	T3 = 1
	**SC**	SC = 1	SC = 1	SC = 1	SC = 1

We then created a categorical outcome variable equal to 2 for readmission, 1 for ED/Obs visit without a readmission, and 0 for no-return to hospital (reference), and formulated our multinomial logistic (mLogit) difference-in-differences model the following way:

$mLogit\ [P(y=1),P(y=2)] = \text{regression intercept} + \beta_1 (T1) + \beta_2 (T2) + \beta_3 (T3) + \beta_4 (SC) + \beta_5 [(T1)\times(SC)] + \beta_6 [(T2)\times(SC)] + \beta_7 [(T3)\times(SC)] + X'b + h'\varsigma.$ *[2]*

The multinomial logistic regression simultaneously estimates two equations (one for readmission and one for ED/Obs, each relative to the no-outcome baseline), while automatically adjusting for correlated errors and multiple hypothesis testing. In addition to patient controls, our study model includes 33 fixed effects for the study hospitals $h'\varsigma$. The study model is adjusted for clustering at the hospital and unit level.

In both of the estimated outcome equations, β_5, β_6, and β_7 are the difference-in-differences effect estimates that reflect the change in readmissions and ED/Obs visits caused by each of the three protocols on the intervention units. After estimating the log-transformed model, we derived the average predictive margins[3] for each of the difference-

© 2019 Weiss ME et al. *JAMA Network Open.*

in-differences estimated for each protocol and tested these margins for significance using two-sided Wald tests at the standard 5% significance level.

eREFERENCES

1. Imbens GW, Rubin DB. Causal inference for statistics, social and biomedical sciences. Cambridge, UK: Cambridge University Pres;. 2015.
2. Dimick JB, Ryan AM. Methods for evaluating changes in health care policy: The difference-in-differences approach, *JAMA*. 2014; 312(22): 2401-2402.
3. Williams R. Using the margins command to estimate and interpret adjusted predictions and marginal effects. *The Stata Journal*. 2012;12(2):308–331.

© 2019 Weiss ME et al. *JAMA Network Open.*

Effect of Implementing Discharge Readiness Assessment in Adult Medical-Surgical Units on 30-day Return to Hospital: The READI Randomized Clinical Trial

Weiss ME, Yakusheva O, Bobay KL, Costa L, Hughes RG, Nuccio S, Bahr S, Siclovan D, Hamilton M, Bang J.

eTables

eTable 1. Difference-in-Differences estimates of the effect of the READI intervention on 30-day post-discharge return to hospital: Full model

	READI1[a]				READI2[b]				READI3[c]			
	Intervention	Control	Difference		Intervention	Control	Difference		Intervention	Control	Difference	
	Adjusted Rate[d] (95% CI)	Adjusted Rate[d] (95% CI)	Intervention - Control (95% CI)	P	Adjusted Rate[d] (95% CI)	Adjusted Rate[d] (95% CI)	Intervention - Control (95% CI)	P	Adjusted Rate[d] (95% CI)	Adjusted Rate[d] (95% CI)	Intervention - Control (95% CI)	P
**All Units**												
**ITT**												
**Readmission**	12.94	11.92	1.02	0.03	12.23	12.43	-0.20	0.66	12.23	11.64	0.59	0.22
	(12.14 14.75)	(11.46 12.39)	(0.10 1.90)		(11.46 12.99)	(1.96 12.90)	(-1.10 0.70)		(11.76 12.69)	(11.14 12.13)	(-0.30 1.50)	
**ED/Obs**	8.45	9.60	-1.15	0.01	8.39	8.69	-0.29	0.47	8.71	9.35	-0.64	0.14
	(7.76 9.15)	(9.18 10.20)	(-2.00 -0.30)		(7.69 9.10)	(8.29 9.09)	(-1.10 0.50)		(8.29 9.14)	(8.90 9.79)	(-1.50 0.20)	
**TPP**												
**Readmission**	12.27	11.83	0.44	0.46	11.32	12.45	-1.13	0.08	12.26	11.63	0.63	0.25
	(11.11 13.42)	(11.20 12.46)	(-0.80 1.60)		(10.54 12.10)	(11.58 13.31)	(-2.40 0.2)		(11.28 13.25)	(10.91 12.35)	(-0.50 1.70)	
**ED/Obs**	8.53	9.64	-1.12	0.07	7.99	8.72	-0.73	0.21	8.60	9.44	-0.84	0.10
	(7.79 9.26)	(8.84 10.45)	(-2.30 0.10)		(7.24 8.74)	(8.30 9.14)	(-1.90 0.40)		(8.01 9.19)	(8.90 9.98)	(-1.90 0.20)	

© 2019 Weiss ME et al. *JAMA Network Open.*

	READI1[a]				READI2[b]				READI3[c]			
	Intervention Adjusted Rate[d] (95% CI)	Control Adjusted Rate[d] (95% CI)	Difference Intervention - Control (95% CI)	P	Intervention Adjusted Rate[d] (95% CI)	Control Adjusted Rate[d] (95% CI)	Difference Intervention - Control (95% CI)	P	Intervention Adjusted Rate[d] (95% CI)	Control Adjusted Rate[d] (95% CI)	Difference Intervention - Control (95% CI)	P
**Unit Baseline Readmission Rate <=11.3%**												
**ITT**												
**Readmission**	12.22	9.63	2.60	<0.001	10.39	8.98	1.41	0.05	10.16	9.37	0.79	0.26
	(10.71 13.74)	(8.80 10.45)	(1.40 3.80)		(9.56 11.21)	(8.11 9.84)	(0.00 2.80)		(9.01 11.30)	(8.62 10.12)	(-0.60 2.20)	
**ED/Obs**	7.02	9.35	-2.33	<0.001	7.55	8.87	-1.32	0.02	8.06	9.48	-1.43	0.02
	(6.26 7.77)	(8.61 10.09)	(-3.50 -1.20)		(6.67 8.42)	(8.25 9.48)	(-2.40 -0.20)		(7.46 8.65)	(8.72 10.25)	(-2.70 -0.20)	
**TPP**												
**Readmission**	11.54	9.53	2.01	0.02	9.82	9.03	0.79	0.30	10.18	9.38	0.80	0.32
	(10.07 13.01)	(8.79 10.28)	(0.40 3.60)		(8.94 10.70)	(8.25 9.81)	(-0.80 2.30)		(8.85 11.51)	(8.61 10.14)	(-0.80 2.40)	
**ED/Obs**	7.23	9.39	-2.16	<0.001	7.27	8.97	-1.69	0.008	8.01	9.62	-1.60	0.04
	(6.29 8.17)	(8.70 10.08)	(-3.20 -1.10)		(6.40 8.15)	(8.36 9.58)	(-3.00 -0.40)		(7.26 8.77)	(8.82 10.41)	(-3.10 -0.10)	
**Unit Baseline Readmission Rate >11.3%**												
**ITT**												
**Readmission**	13.62	14.33	-0.71	0.17	14.18	15.97	-1.79	0.009	14.39	14.00	0.39	0.60
	(12.38 14.85)	(13.27 15.39)	(-1.70 0.30)		(13.41 14.95)	(14.67 17.26)	(-3.20 -0.40)		(13.08 15.70)	(12.72 15.29)	(-1.10 1.90)	
**ED/Obs**	10.03	9.82	0.21	0.81	9.33	8.48	0.86	0.26	9.41	9.20	0.21	0.65
	(9.15 10.91)	(8.47 11.17)	(-1.50 1.90)		(8.15 10.52)	(7.89 9.06)	(-0.70 2.40)		(8.71 10.11)	(8.48 9.92)	(-0.70 1.10)	

© 2019 Weiss ME et al. *JAMA Network Open.*

	READI1[a]				READI2[b]				READI3[c]			
	Intervention Adjusted Rate[d] (95% CI)	Control Adjusted Rate[d] (95% CI)	Difference Intervention - Control (95% CI)	P	Intervention Adjusted Rate[d] (95% CI)	Control Adjusted Rate[d] (95% CI)	Difference Intervention - Control (95% CI)	P	Intervention Adjusted Rate[d] (95% CI)	Control Adjusted Rate[d] (95% CI)	Difference Intervention - Control (95% CI)	P
**TPP**												
**Readmission**	12.89	14.28	-1.38	0.02	12.89	15.94	-3.05	<0.001	14.46	13.99	0.47	0.50
	(11.41 14.37)	(13.31 15.25)	(-2.50 -0.30)		(12.13 13.66)	(14.64 17.24)	(-4.50 -1.60)		(13.05 15.86)	(12.80 15.18)	(-0.90 1.90)	
**ED/Obs**	10.00	9.88	0.12	0.90	8.79	8.45	0.35	0.68	9.20	9.25	-0.06	0.91
	(8.98 11.03)	(8.45 11.32)	(-1.90 2.10		(7.69 9.90)	(7.90 8.99)	(-1.40 2.10)		(8.30 10.10)	(8.55 9.96)	(-1.10 0.90)	

Abbreviations: "Difference." = fully adjusted difference between intervention (I) and control units (C); "Readmission" = Inpatient readmission; ED/Obs" = Emergency Department / Observation visit; "ITT" = Intent to treat; "TPP" = treated per protocol.

a READI1: Readiness Evaluation And Discharge Interventions Protocol 1: Nurse assessment using nurse form of the Readiness for Hospital Discharge Scale with instruction to nurses to use their best judgment with the assessment information to guide actions in completing their patients' preparation for discharge .

b READI2: Readiness Evaluation And Discharge Interventions Protocol 2: Patient self-assessment using the patient form of the Readiness for Hospital Discharge Scale followed by nurse assessment using the nurse form of the Readiness for Hospital Discharge Scale with instruction to nurses to use their best judgment with the assessment information to guide actions in completing their patients' preparation for discharge .

c READI3: Readiness Evaluation And Discharge Interventions Protocol 3: Patient self-assessment using the patient form of the Readiness for Hospital Discharge Scale followed by nurse assessment using the nurse form of the Readiness for Hospital Discharge Scale with instruction to nurses to act and document nurse actions if low readiness score (<7) on nurse or patient form.

d Adjusted for baseline and patient characteristics. Rates expressed per 100 index patient discharges. Adjusted rates and adjusted difference in rates were estimated using a multinomial logistic regression with adjustment for baseline event rates and patient characteristics and clustering at unit and hospital level.

≠ ED/Obs: Emergency Department visit or Observation stay with no record of readmission. ED and Observation were combined as return to hospital without inpatient readmission.

© 2019 Weiss ME et al. *JAMA Network Open.*

**eTable 2. Difference-in-Differences estimates of the effect of the READI intervention on 60-day post-discharge return to hospital: Full model**												
	**READI1[a]**				**READI2[b]**				**READI3[c]**			
	**Intervention**	**Control**	**Difference**		**Intervention**	**Control**	**Difference**		**Intervention**	**Control**	**Difference**	
	**Adjusted Rate[d] (95% CI)**	**Adjusted Rate[d] (95% CI)**	**Intervention - Control (95% CI)**	**P**	**Adjusted Rate[d] (95% CI)**	**Adjusted Rate[d] (95% CI)**	**Intervention - Control (95% CI)**	**P**	**Adjusted Rate[d] (95% CI)**	**Adjusted Rate[d] (95% CI)**	**Intervention - Control (95% CI)**	**P**
**All**												
**ITT**												
**Readmission**	17.67	17.26	0.41	0.50	17.17	17.41	-0.25	0.72	17.08	16.55	0.53	0.32
	(16.51 18.84)	(16.50 18.02)	(-0.80 1.60)		(16.31 18.02)	(16.39 18.43)	(-1.60 1.10)		(16.09 18.07)	(15.75 17.34)	(-0.60 1.60)	
**ED/Obs**	10.53	11.68	-1.15	0.10	10.41	10.89	-0.47	0.41	10.91	11.32	-0.41	0.51
	(9.81 11.26)	(10.82 12.84)	(-2.50 0.20)		(9.45 11.37)	(10.41 11.37)	(-1.60 0.70)		(10.38 11.43)	(10.72 11.92)	(-1.70 0.90)	
**TPP**												
**Readmission**	17.67	17.26	0.41	0.50	17.17	17.41	-0.25	0.72	17.08	16.55	0.53	0.32
	(16.51 18.84)	(16.50 18.02)	(-0.80 1.60)		(16.31 18.02)	(16.39 18.43)	(-1.60 1.10)		(16.09 18.07)	(15.75 17.34)	(-0.60 1.60)	
**ED/Obs**	10.53	11.68	-1.15	0.10	10.41	10.89	-0.47	0.41	10.91	11.32	-0.41	0.51
	(9.81 11.26)	(10.82 12.84)	(-2.50 0.20)		(9.45 11.37)	(10.41 11.37)	(-1.60 0.70)		(10.38 11.43)	(10.72 11.92)	(-1.70 0.90)	
**Unit Baseline Readmission Rate <=11.3%**												
**ITT**												
**Readmission**	17.67	17.26	0.41	0.50	17.17	17.41	-0.25	0.72	17.08	16.55	0.53	0.32
	(16.51 18.84)	(16.50 18.02)	(-0.80 1.60)		(16.31 18.02)	(16.39 18.43)	(-1.60 1.10)		(16.09 18.07)	(15.75 17.34)	(-0.60 1.60)	
**ED/Obs**	10.53	11.68	-1.15	0.10	10.41	10.89	-0.47	0.41	10.91	11.32	-0.41	0.51
	(9.81 11.26)	(10.82 12.84)	(-2.50 0.20)		(9.45 11.37)	(10.41 11.37)	(-1.60 0.70)		(10.38 11.43)	(10.72 11.92)	(-1.70 0.90)	

© 2019 Weiss ME et al. *JAMA Network Open.*

	READI1[a]				READI2[b]				READI3[c]			
	Intervention Adjusted Rate[d] (95% CI)	Control Adjusted Rate[d] (95% CI)	Difference Intervention - Control (95% CI)	P	Intervention Adjusted Rate[d] (95% CI)	Control Adjusted Rate[d] (95% CI)	Difference Intervention - Control (95% CI)	P	Intervention Adjusted Rate[d] (95% CI)	Control Adjusted Rate[d] (95% CI)	Difference Intervention - Control (95% CI)	P
**TPP**												
**Readmission**	15.23	13.89	1.35	0.19	14.09	13.24	0.85	0.37	14.10	13.29	0.82	0.31
	(13.47 17.00)	(13.25 14.52)	(-0.70 3.40)		(13.06 15.11)	(12.25 14.43)	(-1.10 2.80)		(12.39 15.82)	(12.48 14.09)	(-0.80 2.40)	
**ED/Obs**	9.50	11.11	-1.62	0.04	9.81	11.04	-1.23	0.10	10.29	11.12	-0.84	0.39
	(8.58 10.42)	(10.22 12.01)	(-3.10 -0.10)		(8.75 10.88)	(10.45 11.62)	(-2.70 0.20)		(9.33 11.24)	(10.44 11.81)	(-2.80 1.10)	
**Unit Baseline Readmission Rate >11.3%**												
**ITT**												
**Readmission[f]**	19.18	20.66	-1.48	0.02	19.78	21.81	-2.03	0.02	20.32	19.96	0.36	0.62
	(17.82 20.53)	(19.41 21.91)	(-2.70 -0.20)		(18.99 2057)	(20.32 23.30)	(-3.70 -0.4)		(18.97 21.67)	(18.59 21.33)	(-1.10 1.90)	
**ED/Obs**	11.91	12.29	-0.38	0.69	10.88	10.86	0.02	0.98	11.39	11.67	-0.28	0.74
	(10.86 12.96)	(10.89 13.67)	(-2.30 1.60)		(9.36 12.40)	(10.17 11.54)	(-1.60 1.70)		(10.79 12.00)	(10.72 12.63)	(-2.00 1.40)	
**TPP**												
**Readmission**	18.39	20.63	-2.24	0.003	18.13	21.76	-3.63	<0.001	20.22	19.96	0.26	0.71
	(16.43 20.36)	(19.53 21.73)	(-3.70 -0.80)		(17.40 18.86)	(20.24 23.29)	(-5.10 -2.20)		(18.76 21.68)	(18.75 21.16)	(-1.20 1.70)	
**ED/obs**	11.85	12.35	-0.50	0.65	10.54	10.80	-0.26	0.77	11.52	11.69	-0.17	0.84
	(10.54 13.15)	(10.85 13.85)	(-2.70 1.70)		(9.07 12.01)	(10.14 11.47)	(-2.10 1.60)		(10.62 12.42)	(10.78 12.59)	(-1.90 1.50)	

© 2019 Weiss ME et al. *JAMA Network Open.*

Abbreviations: “Difference.” = fully adjusted difference between intervention (I) and control units (C); “Readmission” = Inpatient readmission;
ED/Obs” = Emergency Department / Observation visit; "ITT" = Intent to treat; "TPP" = treated per protocol.
a READI1: Readiness Evaluation And Discharge Interventions Protocol 1: Nurse assessment using nurse form of the Readiness for Hospital Discharge Scale with instruction to nurses to use their best judgment with the assessment information to guide actions in completing their patients’ preparation for discharge .
b READI2: Readiness Evaluation And Discharge Interventions Protocol 2: Patient self-assessment using the patient form of the Readiness for Hospital Discharge Scale followed by nurse assessment using the nurse form of the Readiness for Hospital Discharge Scale with instruction to nurses to use their best judgment with the assessment information to guide actions in completing their patients’ preparation for discharge .
c READI3: Readiness Evaluation And Discharge Interventions Protocol 3: Patient self-assessment using the patient form of the Readiness for Hospital Discharge Scale followed by nurse assessment using the nurse form of the Readiness for Hospital Discharge Scale with instruction to nurses to act and document nurse actions if low readiness score (<7) on nurse or patient form.
d Adjusted for baseline and patient characteristics. Rates expressed per 100 index patient discharges. Adjusted rates and adjusted difference in rates were estimated using a multinomial logistic regression with adjustment for baseline event rates and patient characteristics and clustering at unit and hospital level.
[≠] ED/Obs: Emergency Department visit or Observation stay with no record of readmission. ED and Observation were combined as return to hospital without inpatient readmission.

© 2019 Weiss ME et al. *JAMA Network Open.*

eTable 3. Robustness of 30-day results, without Saudi Hospitals (ITT n=137,814; TPP n=121,763)									
	ALL			High-readmission units			Low-readmission units		
	READI1[a]	READI2[b]	READI3[c]	READI1[a]	READI2[b]	READI3[c]	READI1[a]	READI2[b]	READI3[c]
	Difference (95% CI) *P*	Difference (95% CI) *P*	Difference (95% CI) *P*	Difference (95% CI) *P*	Difference (95% CI) *P*	Difference (95% CI) *P*	Difference (95% CI) *P*	Difference (95% CI) *P*	Difference (95% CI) *P*
**ITT**									
**Readmission**	0.91	-0.28	0.49	-0.71	-1.79	0.38	2.53	1.38	0.62
	(-0.13 1.95)	(-1.43 0.86)	(-0.58 1.56)	(-1.73 0.32)	(-3.19 0.39)	(-1.12 1.88)	(1.19 3.87)	(-0.12 2.89)	(-0.89 2.14)
	0.08	*0.62*	*0.36*	*0.17*	*0.01**	*0.61*	*<0.001***	*0.07*	*0.41*
**ED/Obs**	-0.90	-0.04	-0.31	0.22	0.85	0.24	-2.00	-0.92	-0.87
	(-2.03 0.24)	(-1.08 0.99)	(-1.12 0.51)	(-1.46 1.91)	(-0.70 2.39)	(-0.68 1.15)	(-3.26 -0.74)	(-2.02 0.19)	(-2.06 0.32)
	0.12	*0.93*	*0.45*	*0.79*	*0.27*	*0.6*	*0.002***	*0.10*	*0.55*
**TPP**									
**Readmission**	0.30	-1.24	0.48	-1.38	-3.03	0.46	1.93	0.73	0.52
	(-0.94 1.54)	(-2.59 0.11)	(-0.63 1.59)	(-2.51 - 0.25)	(-4.50 -1.57)	(-0.93 1.84)	(0.14 3.73)	(-0.99 2.45)	(-1.21 2.24)
	0.62	*0.07*	*0.38*	*0.02**	*<0.001***	*0.50*	*0.04**	*0.39*	*0.55*
**ED/Obs.**	-0.87	-0.49	-0.48	0.13	0.34	-0.03	-1.83	-1.31	-0.95
	(-2.09 0.36)	(-1.68 0.69)	(-1.45 0.48)	(-1.88 2.15)	(-1.38 2.06)	(-1.04 0.99)	(-2.98 -0.69)	(-2.70 0.07)	(-2.46 0.59)
	0.16	*0.40*	*0.31*	*0.89*	*0.69*	*0.96*	*0.002***	*0.06*	*0.22*

Abbreviations: "Difference." = fully adjusted difference between intervention (I) and control units (C); "Readmission" = Inpatient readmission; ED/Obs" = Emergency Department / Observation visit; "ITT" = Intent to treat; "TPP" = treated per protocol.
a READI1: Readiness Evaluation And Discharge Interventions Protocol 1: Nurse assessment using nurse form of the Readiness for Hospital Discharge Scale with instruction to nurses to use their best judgment with the assessment information to guide actions in completing their patients' preparation for discharge .
b READI2: Readiness Evaluation And Discharge Interventions Protocol 2: Patient self-assessment using the patient form of the Readiness for Hospital Discharge Scale followed by nurse assessment using the nurse form of the Readiness for Hospital Discharge Scale with instruction to nurses to use their best judgment with the assessment information to guide actions in completing their patients' preparation for discharge .
c READI3: Readiness Evaluation And Discharge Interventions Protocol 3: Patient self-assessment using the patient form of the Readiness for Hospital Discharge Scale followed by nurse assessment using the nurse form of the Readiness for Hospital Discharge Scale with instruction to nurses to act and document nurse actions if low readiness score (<7) on nurse or patient form.
d Adjusted for baseline and patient characteristics. Rates expressed per 100 index patient discharges. Adjusted rates and adjusted difference in rates were estimated using a multinomial logistic regression with adjustment for baseline event rates and patient characteristics and clustering at unit and hospital level.
≠ ED/Obs: Emergency Department visit or Observation stay with no record of readmission. ED and Observation were combined as return to hospital without inpatient readmission.

© 2019 Weiss ME et al. *JAMA Network Open.*

eTable 4. Robustness of 30-day results for units with a formal discharge model of care in place prior to READI study (ITT n=73,866; TPP n=53,187)

	ALL			High-readmission units			Low-readmission units		
	READI1[a]	READI2[b]	READI3[c]	READI1[a]	READI2[b]	READI3[c]	READI1[a]	READI2[b]	READI3[c]
	Difference (95% CI) *P*	Difference (95% CI) *P*	Difference (95% CI) *P*	Difference (95% CI) *P*	Difference (95% CI) *P*	Difference (95% CI) *P*	Difference (95% CI) *P*	Difference (95% CI) *P*	Difference (95% CI) *P*
**ITT**									
**Readmission**	1.19	0.11	-0.16	-1.4	-1.79	-1.36	2.85	1.41	0.63
	(-0.41 2.78)	(-1.78 2.01)	(-2.04 1.72)	(-3.06 0.27)	(-4.71 1.12)	(-4.3 1.58)	(1.24 4.46)	(-0.28 3.31)	(-1.68 2.95)
	0.14	*0.9*	*0.86*	*0.10*	*0.21*	*0.34*	*0.001***	*0.10*	*0.57*
**ED/Obs**	-1.17	0.04	-0.56	1.35	1.99	0.73	-2.79	-1.24	-1.44
	(-2.86 0.53)	(-1.19 1.27)	(-1.98 0.86)	(-0.60 3.31)	(0.60 3.37)	(-1.12 2.59)	(-4.52 -1.06)	(-2.45 -0.04)	(-3.04 0.16)
	0.17	*0.95*	*0.42*	*0.16*	*0.005***	*0.42*	*0.002***	*0.04**	*0.07*
**TPP**									
**Readmission**	0.36	-1.04	0.11	-2.46	-3.44	-0.63	2.13	0.59	0.59
	(-1.63 2.36)	(-2.99 0.90)	(-1.84 2.08)	(-4.00 -0.93)	(-5.59 -1.3)	(-3.31 2.04)	(-0.25 4.52)	(-1.68 2.86)	(-2.03 3.21)
	0.71	*0.27*	*0.91*	*0.002***	*0.004***	*0.62*	*0.08*	*0.59*	*0.64*
**ED/Obs**	-0.93	-0.37	-0.83	1.45	1.32	0.53	-2.45	-1.51	-1.77
	(-2.62 0.76)	(-1.66 0.92)	(-2.40 0.73)	(-0.73 3.62)	(0.11 2.54)	(-1.16 2.21)	(-4.14 -0.76)	(-3.04 0.02)	-3.63 0.08)
	0.26	*0.55*	*0.28*	*0.18*	*0.03**	*0.52*	*0.005***	*0.05*	*0.06*

Abbreviations: "Difference." = fully adjusted difference between intervention (I) and control units (C); "Readmission" = Inpatient readmission; ED/Obs" = Emergency Department / Observation visit; "ITT" = Intent to treat; "TPP" = treated per protocol.

a READI1: Readiness Evaluation And Discharge Interventions Protocol 1: Nurse assessment using nurse form of the Readiness for Hospital Discharge Scale with instruction to nurses to use their best judgment with the assessment information to guide actions in completing their patients' preparation for discharge .

b READI2: Readiness Evaluation And Discharge Interventions Protocol 2: Patient self-assessment using the patient form of the Readiness for Hospital Discharge Scale followed by nurse assessment using the nurse form of the Readiness for Hospital Discharge Scale with instruction to nurses to use their best judgment with the assessment information to guide actions in completing their patients' preparation for discharge .

c READI3: Readiness Evaluation And Discharge Interventions Protocol 3: Patient self-assessment using the patient form of the Readiness for Hospital Discharge Scale followed by nurse assessment using the nurse form of the Readiness for Hospital Discharge Scale with instruction to nurses to act and document nurse actions if low readiness score (<7) on nurse or patient form.

d Adjusted for baseline and patient characteristics. Rates expressed per 100 index patient discharges. Adjusted rates and adjusted difference in rates were estimated using a multinomial logistic regression with adjustment for baseline event rates and patient characteristics and clustering at unit and hospital level.

≠ ED/Obs: Emergency Department visit or Observation stay with no record of readmission. ED and Observation were combined as return to hospital without inpatient readmission.

© 2019 Weiss ME et al. *JAMA Network Open.*

eTable 5. Robustness of 30-day results for units without a formal discharge model of care in place prior to READI study (ITT n=71,002; TPP n=56,252)

	ALL			High-readmission units			Low-readmission units		
	READI1[a]	READI2[b]	READI3[c]	READI1[a]	READI2[b]	READI3[c]	READI1[a]	READI2[b]	READI3[c]
	Difference (95% CI) *P*	Difference (95% CI) *P*	Difference (95% CI) *P*	Difference (95% CI) *P*	Difference (95% CI) *P*	Difference (95% CI) *P*	Difference (95% CI) *P*	Difference (95% CI) *P*	Difference (95% CI) *P*
**ITT**									
**Readmission**	0.72	-1.04	1.23	-0.26	-2.14	1.55	2.06	0.59	0.85
	(-0.71 2.15)	(-2.52 0.44)	(0.04 2.42)	(-1.75 1.23)	(-3.82 -0.47)	(0.14 2.95)	(0.00 4.12)	(-1.27 2.45)	(-0.99 2.68)
	0.30	*0.16*	*0.04**	*0.72*	*0.02**	*0.03**	*0.05**	*0.51*	*0.34*
**ED/Obs**	-0.97	-0.24	-0.51	-0.44	0.31	0.08	-1.74	-1.11	-1.39
	(-2.6 0.66)	(-1.92 1.43)	(-1.83 0.81)	(-2.61 1.73)	(-2.08 2.71)	(-1.09 1.24)	(-3.42 -0.06)	(-3.24 1.02)	(-3.6 0.82)
	0.23	*0.76*	*0.42*	*0.67*	*0.78*	*0.89*	*0.04**	*0.29*	*0.20*
**TPP**									
**Readmission**	0.5	-1.4	1.13	-0.63	-2.79	1.11	1.85	0.64	1.14
	(-1.16 2.16)	(-3.02 0.22)	(-0.33 2.57)	(-2.49 1.22)	(-4.85 -0.72)	(-0.76 2.99)	(-0.31 4.01)	(-1.14 2.41)	-0.94 3.21)
	0.53	*0.09*	*0.12*	*0.48*	*0.01**	*0.23*	*0.09*	*0.46*	*0.26*
**ED/Obs**	-1.23	-0.73	-0.57	-0.7	-0.09	-0.25	-1.87	-1.65	-1.17
	(-3.16 0.69)	(-2.67 1.21)	(-2.15 1.00)	(-3.49 2.08)	(-2.98 2.81)	(-1.70 1.20)	(-3.51 -0.23)	(-3.98 0.68)	(-4.04 1.71)
	0.19	*0.44*	*0.45*	*0.6*	*0.95*	*0.72*	*0.03**	*0.15*	*0.4*

Abbreviations: "Difference." = fully adjusted difference between intervention (I) and control units (C); "Readmission" = Inpatient readmission; ED/Obs" = Emergency Department / Observation visit; "ITT" = Intent to treat; "TPP" = treated per protocol.
a READI1: Readiness Evaluation And Discharge Interventions Protocol 1: Nurse assessment using nurse form of the Readiness for Hospital Discharge Scale with instruction to nurses to use their best judgment with the assessment information to guide actions in completing their patients' preparation for discharge .
b READI2: Readiness Evaluation And Discharge Interventions Protocol 2: Patient self-assessment using the patient form of the Readiness for Hospital Discharge Scale followed by nurse assessment using the nurse form of the Readiness for Hospital Discharge Scale with instruction to nurses to use their best judgment with the assessment information to guide actions in completing their patients' preparation for discharge .
c READI3: Readiness Evaluation And Discharge Interventions Protocol 3: Patient self-assessment using the patient form of the Readiness for Hospital Discharge Scale followed by nurse assessment using the nurse form of the Readiness for Hospital Discharge Scale with instruction to nurses to act and document nurse actions if low readiness score (<7) on nurse or patient form.
d Adjusted for baseline and patient characteristics. Rates expressed per 100 index patient discharges. Adjusted rates and adjusted difference in rates were estimated using a multinomial logistic regression with adjustment for baseline event rates and patient characteristics and clustering at unit and hospital level.
≠ ED/Obs: Emergency Department visit or Observation stay with no record of readmission. ED and Observation were combined as return to hospital without inpatient readmission.

© 2019 Weiss ME et al. *JAMA Network Open.*
