# Supplementary material for: Effect of Implementing Discharge Readiness Assessment in Adult Medical-Surgical Units on 30-Day Return to Hospital: The READI Randomized Clinical Trial
Source: JAMA Netw Open. 2019 Jan 25;2(1):e187387. doi: 10.1001/jamanetworkopen.2018.7387 (PMC6484543; doi:10.1001/jamanetworkopen.2018.7387)
Supplement: Supplement 3. — Data Sharing Statement [file jamanetwopen-2-e187387-s003.pdf]

Data Sharing Statement

Weiss. Effect of Implementing Discharge Readiness Assessment in Adult Medical-Surgical Units on 30-Day Return to Hospital. *JAMA Netw Open*. Published January 25, 2019. 10.1001/jamanetworkopen.2018.7387

Data
Data available: No

Additional Information
Explanation for why data not available: Data provided to researchers by hospital sites cannot be shared.
